# Nationwide trends in childhood cancer incidence and survival in Iran: analysis of national cancer registry data, 2005–2014

**DOI:** 10.1186/s12887-025-06363-4

**Published:** 2025-12-05

**Authors:** Mehdi Azizmohammad Looha, Ali Saberi Shahrbabaki, Mohammad Esmaeil Akbari, Azin Mohammadpoor, Soheila Khodakarim

**Affiliations:** 1 Pediatric Pathology Research Center, Research Institute for Children’s Health, Shahid Beheshti University of Medical Sciences, Tehran, Iran; 2 Basic and Molecular Epidemiology of Gastrointestinal Disorders Research Center, Research Institute for Gastroenterology and Liver Diseases, Shahid Beheshti University of Medical Sciences, Tehran, Iran; 3 School of Medicine, Shahid Beheshti University of Medical Sciences, Tehran, Iran; 4 Cancer Research Center, Shahid Beheshti University of Medical Sciences, Tehran, Iran; 5 King George Hospital, London, England; 6 Department of Biostatistics, School of Medicine, Shiraz University of Medical Sciences, Shiraz, Iran

**Keywords:** Neoplasms, Pediatrics, Incidence, Survival, Iran, Registries, Leukemia, Lymphoma, Central nervous system

## Abstract

**Background:**

Childhood cancer poses a growing global health burden. This study aimed to assess incidence patterns, temporal trends and five-year survival probabilities of childhood cancers in Iran using national cancer registry data from 2005 to 2014.

**Methods:**

A retrospective analysis was conducted using data from the Iran National Cancer Registry. After quality control procedures, 20,624 validated cases of childhood cancer (ages 0–14 years) were included. Age-standardized incidence rates (ASIRs) were calculated per million person-years using the new World Health Organization standard population. Trends over time were examined, and five-year observed survival probabilities were estimated for major diagnostic categories according to the International Classification of Childhood Cancer, Third Edition.

**Results:**

The overall ASIR for childhood cancers in Iran was 116.2 per million person-years, higher in males (128.1) than in females (103.6). The most common cancer groups were leukemia (ASIR: 31.7), lymphomas (ASIR: 13.2), and central nervous system neoplasms (ASIR: 12.6). Lymphoid leukemia was the most frequent subtype (ASIR: 26.6 in males, 20.0 in females). Joinpoint analysis indicated a modest but significant increase in overall ASIRs in both sexes (average annual percent change ≈ 3.5%), driven mainly by central nervous system and hepatic tumors, while retinoblastoma and Hodgkin lymphoma declined. The overall five-year survival was 81.0%, improving from 70.2% in 2005–2009 to 81.4% in 2010–2014. The highest survival was observed in lymphomas (97.1%), followed by retinoblastoma (96.9%), and soft tissue sarcomas (90.7%), while the lowest was found in neuroblastoma (39.1%). Children aged 0–4 years had the lowest survival (79.6%), compared with 82.9% and 82.0% in those aged 5–9 and 10–14 years, respectively.

**Conclusions:**

This nationwide study presents a comprehensive analysis of childhood cancer incidence and survival in Iran. The findings indicate a gradual increase in incidence, particularly among boys and younger children. Survival appears to have improved over time, though variations by age and cancer type warrant cautious interpretation due to differences in follow-up and case mix. These findings highlight progress in pediatric oncology in Iran and underscore the importance of strengthening cancer registration, diagnostic capacity, and equitable access to specialized care to further improve outcomes for children with cancer.

**Supplementary Information:**

The online version contains supplementary material available at 10.1186/s12887-025-06363-4.

## Introduction

Cancer remains a leading cause of death among children worldwide, with an estimated 206,362 new cases and 80,104 deaths in 2020 among those aged 0–14 years [1]. Childhood cancers are typically fatal without early diagnosis and treatment [2]. They account for about 1% of all cancers in high-income countries but a much higher share in developing regions [3]. In Iran, a meta-analysis estimated an incidence rate of around 170 per million among children aged 0–14 years [4]. According to the International Classification of Childhood Cancer, Third Edition (ICCC-3), most childhood cancers fall into three major categories including leukemia, central nervous system (CNS) tumors, and lymphomas [5, 6]. Over 80% of these cancers occur in low- and middle-income countries such as Iran, Egypt, and India [1, 7, 8].

The age-standardized incidence rate (ASIR) of childhood cancer varies widely across countries, largely reflecting Please check if affiliations [is/are] captured correctly.differences in diagnostic capacity, registry completeness, and healthcare access rather than true etiological variation. Limited studies have explored genetic or environmental factors, but evidence remains inconsistent [1, 3, 9]. Countries with higher Human Development Index (HDI) values generally report higher ASIRs, likely due to better registration systems, diagnostic accuracy, and awareness [4, 10, 11]. In contrast, lower rates in low-HDI settings are more plausibly explained by underdiagnosis and incomplete reporting [1]. Globally, childhood cancer ASIRs have increased in recent decades, mainly due to improved registry coverage and data quality rather than real rises in risk [12]. Despite numerous investigations, most childhood cancers have unclear causes and are believed to arise from complex, multifactorial processes, highlighting the need for continued epidemiological research [13–17].

In developing countries such as Iran, monitoring trends in childhood cancer remains challenging due to limited, population-based data and incomplete registry coverage. An earlier national study in Iran reported wide variation in ASIR estimates, reflecting inconsistencies in data sources and quality [18]. However, unlike the prior study [19], the present study aimed to assess the incidence and temporal trends of childhood cancers among Iranian children aged 0–14 years during 2005–2014 by ICCC-3 subgroups, evaluate five-year survival probabilities, and examine provincial variations using data from the National Cancer Registry.

## Materials and methods

### Study design

This was a descriptive, retrospective cohort study utilizing data from the Iran National Cancer Registry (INCR) to investigate the incidence of childhood cancer. The study adhered to standardized reporting practices and was designed to explore trends over a 10-year period (2005–2014).

### Setting

The study was conducted in Iran, using data recorded between March 20, 2005, and March 20, 2014. The INCR, operating under the Ministry of Health and Medical Education, systematically collects and reports cancer data from hospitals, pathology laboratories, and health centers across all provinces. Follow-up information for survival analysis was collected in 2020 through telephone interviews with patients or their families.

### Participants

Eligible participants included all registered cases of childhood cancer (ages 0–14 years) documented in the INCR during the study period. The inclusion criterion was a diagnosis with any cancer categorized under the ICCC-3 [20]. Only malignant primary tumors (behaviour code/3 according to International Classification of Diseases for Oncology, Third Edition [ICD-O-3]) were included, while benign (/0), borderline (/1), in situ (/2), secondary (/6), and unspecified (/9) lesions were excluded. This ensured that non-malignant CNS tumors were not included and that international comparability was maintained. After initial data extraction, non-Iranian cases and duplicate entries were removed based on full matching of key identifiers (e.g., name, sex, father’s name, age, ICCC subgroup), resulting in a final sample size of 20,624 cases. The registry includes diagnoses obtained through clinical procedures, pathology reports, or death certificates.

For the survival analysis, follow-up information was obtained from a subset of the registry cases. Among 20,624 eligible patients, contact information was available for 14,735 (71.4%). To ensure feasibility while maintaining representativeness, a simple random sample comprising 50% of these contactable cases (≈7,368) was selected for active follow-up. Vital status and date of death were ascertained through telephone interviews conducted in 2020 with patients or their families.

### Variables and data sources

The primary outcome variable was the incidence of childhood cancer, classified into 12 main groups and 45 subgroups according to the ICCC-3, based on ICD-O-3 codes. Recorded patient-level variables included sex, age at diagnosis, date of birth, ICCC cancer group, province of residence, and date of diagnosis. A secondary outcome was the five-year survival of children diagnosed with cancer, determined through follow-up telephone interviews with patients or their families to ascertain vital status and date of death. During follow-up telephone interviews, a structured follow-up verification checklist was administered to confirm key patient information and determine vital status. Interviewers verified the patient’s name, sex, and age or date of birth against registry data and asked respondents to confirm the primary cancer diagnosis. In cases where the reported diagnosis differed from the registry record, the registry data were retained after verification and clarification with other family members when possible. For deceased patients, the exact or approximate date of death was recorded, and for living patients, the date of the last confirmed contact was documented. The follow-up interviews were designed to confirm patients’ vital status and record the date of death when applicable. Although some respondents voluntarily reported the cause of death, this information was not consistently verifiable and therefore was excluded from the final analysis. Accordingly, survival estimates in this study reflect overall survival rather than cause-specific survival. This checklist was specifically developed for this study and the English version is provided as Supplementary Table S1.

Data were obtained from the INCR, a mandatory nationwide reporting system under the Ministry of Health and Medical Education that compiles cancer data from hospitals, pathology laboratories, and health centers across all provinces. Diagnostic accuracy and data consistency were ensured through multi-step validation, including checks for tumor–sex compatibility, tumor site–age match, morphology–topography consistency, and cross-verification of diagnoses with reported diagnostic procedures. *Death Certificate Only (DCO)* cases were reviewed and retained only when supported by verified diagnostic evidence.

Population data for incidence rate calculations were obtained from the Statistical Center of Iran (SCI) for census years 2006, 2011, and 2016, with values for the intervening years estimated using interpolated growth rates.

### Bias

To minimize selection and classification bias, quality control procedures were implemented by the INCR and the study authors. Duplicate cases and non-Iranian records were excluded systematically. Furthermore, efforts were made to enhance diagnostic accuracy through registry validation and follow-up phone calls. However, some bias due to underreporting or misclassification in earlier years may remain.

### Study size

No sample size calculation was performed, as this study utilized registry-based data and included all eligible childhood cancer cases recorded in the national registry during the 10-year study period. After data cleaning, a total of 20,624 valid cases were analyzed.

### Statistical analysis

#### Descriptive statistics

Descriptive statistics, including frequencies and percentages, were calculated for factors such as sex, age, five-year period of diagnosis, and type of cancer. The binomial proportion test (for factors with two levels) and chi-square test (for factors with more than two levels) were used to assess significant differences in the distribution of these factors. Crude incidence rates (per million person-years) were calculated for each year from 2005 to 2014. The ASIR was used to assess the distribution of childhood cancer types over the 10-year period. The ASIRs were calculated for ICCC groups using the World Health Organization (WHO) world standard population (2000–2025) as the reference population, which provides a more contemporary and demographically balanced standard for international health analyses [21].

#### Trend analysis and comparison

To evaluate sex differences, the male-to-female ASIR ratio (M/F ratio) was computed for each diagnostic group along with its 95% confidence interval (CI). The ratio and CI were calculated using the following expression:

$$\begin{aligned} &\text{M}/\text{F ratio }= \left({\text{ASIR}}_{\text{m}} / {\text{ASIR}}_{\text{f}}\right) \times \\&\left(95{\% CI}:\text{lower }= \left({\text{ASIR}}_{\text{m}} / {\text{ASIR}}_{\text{f}}\right) \right. \\&\left. \wedge \left(1 -1.96 / \left(\left({\text{ASIR}}_{\text{m}}-{\text{ASIR}}_{\text{f}}\right) \right.\right.\right.\\&\left.\left.\left. / \surd \left({\text{SE}}_{\text{m}}^{2} +{\text{SE}}_{\text{x}}^{2} \right)\right)\right),\text{ upper }=\right. \\&\left. \left({\text{ASIR}}_{\text{m}} / {\text{ASIR}}_{\text{f}}\right)\wedge \left(1+1.96 \right. \right. \\&\left. \left. / \left(\left({\text{ASIR}}_{\text{m}}-{\text{ASIR}}_{\text{f}}\right) / \surd \left({\text{SE}}_{\text{m}}^{2} +{\text{SE}}_{\text{x}}^{2}\right)\right)\right)\right) \end{aligned}$$

where ASIRₘ and ASIRₓ represent the male and female ASIRs, and SEₘ and SE_f_ are their respective standard errors.

Temporal changes between the two study intervals (2005–2009 vs. 2010–2014) were also examined by calculating the ASIR ratio for the second five-year period relative to the first and its 95% CI using a similar approach:

$$\begin{aligned} &\text{Period ratio }= \left({\text{ASIR}}_{2} / {\text{ASIR}}_{1}\right) \\&\times \left(95{\% CI}:\text{lower }= \left({\text{ASIR}}_{2} / {\text{ASIR}}_{1}\right) \right. \\&\left.\wedge \left(1 -1.96 / \left(\left({\text{ASIR}}_{2}-{\text{ASIR}}_{1}\right) \right.\right.\right. \\&\left.\left.\left. / \surd \left({\text{SE}}_{2}^{2} +{\text{SE}}_{1}^{2} \right)\right)\right),\right. \\&\left.\text{ upper }= \left({\text{ASIR}}_{2} / {\text{ASIR}}_{1}\right)\right. \\&\left.\wedge \left(1+1.96 / \left(\left({\text{ASIR}}_{2}-{\text{ASIR}}_{1}\right) \right.\right.\right. \\&\left.\left.\left./ \surd \left({\text{SE}}_{2}^{2} +{\text{SE}}_{1}^{2}\right)\right)\right)\right) \end{aligned}$$

where ASIR₁ and ASIR₂ denote the ASIRs for 2005–2009 and 2010–2014, respectively, and SE₁ and SE₂ their standard errors.

To further assess time trends and detect potential inflection points, Joinpoint regression analysis was conducted. This method identifies statistically significant changes in the slope of the trend by fitting segmented log-linear models to annual ASIRs. Analyses were performed for total childhood cancers and for each major diagnostic subgroup by sex using the Joinpoint Regression Program version 5.2.0 (National Cancer Institute, Bethesda, MD, USA). Given the 10-year observation period (2005–2014), the maximum number of joinpoints was restricted to one, following the program’s default recommendations for time series with 7–11 observations. The best-fitting model was selected using the permutation test with an overall significance level of 0.05 [22].

For each segment, the Annual Percentage Change (APC) was derived as:

$$\text{APC }= 100 \times [\text{exp}(\upbeta ) - 1],$$

where β is the regression coefficient for calendar year. When no joinpoint was detected, a single linear trend was assumed across the entire period. The Average Annual Percentage Change (AAPC) was then calculated as a time-weighted average of all APCs to summarize overall trends [23]. Ninety-five percent CIs were computed to determine statistical significance.

#### Projection

The projection of cancer incidence through 2020 was estimated using structural time series based on the crude incidence rate data through 2005 to 2014 among females and males. The structural time series defined by ${X}_{t} = {m}_{t} + {s}_{t} + {Y}_{t}$ where m_t_ is the trend component, s_t_ is the seasonal component, and Y_t_ is the random component [24]. In this model, the X_t_ is predicted by estimating the deterministic components m_t_ and s_t_ in the hope that Y_t_ will turn out to be a stationary time series. Because cancer incidence is not expected to follow a strong seasonal pattern, the seasonal term was assumed negligible in this context. The model decomposes the observed series into deterministic and stochastic components, providing a smooth representation of long-term changes while minimizing the influence of short-term fluctuations. This projection was included to extend the trend through 2020, as nationwide registry data after 2014 were incomplete or inconsistently reported.

#### Survival analysis

Five-year observed survival probabilities were estimated using the Kaplan–Meier method for children diagnosed with cancer during the study period (2005–2014). Survival probabilities were stratified by age group, sex, and major diagnostic categories according to the ICCC-3. Differences in survival distributions between groups were assessed using the log-rank test, with statistical significance set at *p* < 0.05. Ninety-five percent CIs were reported for survival estimates.

#### Spatial analysis

Finally, spatial analysis of provincial ASIRs was performed to illustrate geographical variations in childhood cancer incidence across Iran. ASIRs were calculated separately for males, females, and the total population in three periods (2005–2009, 2010–2014, and 2005–2014). Provincial ASIRs were mapped using the Geographic Information System (GIS) software in R (version 4.5.0) with the *sf*, *ggplot2*, and *viridis* packages. To ensure valid visual comparisons, color scales for 2005–2009 and 2010–2014 maps were standardized using a common range, while the combined 2005–2014 period was displayed using an independent range due to its broader data distribution.

### Ethical considerations

This study was approved by the Ethics Committee of Shahid Beheshti University of Medical Sciences, Tehran, Iran (IR.SBMU.RICH.REC.1399.027). All registry data were de-identified prior to extraction and analysis to maintain patient confidentiality, and all procedures were conducted in accordance with national regulations and the ethical principles of the Declaration of Helsinki. Because the study population involved minors younger than 16 years, all follow-up telephone interviews were conducted with parents or legal guardians, and informed verbal consent was obtained prior to each interview, following the consent procedures approved by the Ethics Committee. For deceased patients, consent for providing survival information was obtained from the closest available next of kin. The Ethics Committee waived the requirement for written informed consent due to the retrospective registry-based design and the minimal-risk nature of the telephone-based follow-up. Participation in the interviews was entirely voluntary, and calls were immediately discontinued if respondents expressed discomfort or preferred not to continue.

## Results

### Data quality assessment and case selection

From the Iranian National Cancer Registry, 26,379 childhood cancer records diagnosed between 2005 and 2014 were retrieved. After applying quality-control procedures, several records were excluded due to invalid or non-Iranian entries (*n* = 391), duplicate cases (*n* = 1,687; detailed in Table S2 in the Supplementary File), incorrect or inconsistent morphology codes (*n* = 2,075), and tumor–sex or site–age mismatches (*n* = 503). Following these steps, a total of 20,624 unique and validated cases remained for incidence and survival analyses. DCO cases, which comprised 7.12% of all records, were thoroughly reviewed, and all were retained after confirmation of diagnostic consistency (Figure S1 in the Supplementary File).

### Total case counts and demographic characteristics

During the 10-year period from 2005 to 2014, a total of 20,624 new childhood cancer cases aged 0–14 years were recorded nationwide. Of these, 8,976 (43.5%) were females and 11,648 (56.5%) were males. The majority of cases occurred among children aged 0–4 years (52.2%), followed by 5–9 years (24.5%) and 10–14 years (23.3%). When stratified by diagnostic period, 8,964 cases (43.5%) were registered during 2005–2009, and 11,660 cases (56.5%) during 2010–2014, indicating a modest upward trend in case ascertainment over time. These distributions and corresponding significance levels are presented in Table 1, where all comparisons were statistically significant at *P* < 0.001.

**Table 1:** Distribution of childhood cancer cases by sex, age group, and diagnostic period in Iran (2005–2014)

Factor	No. of new cases	Percent of new cases	*P*-value
**Sex**			< 0.001^*^
Female	8976	43.5	
Male	11648	56.5	
**Age**			< 0.001^**^
0–4	10773	52.2	
5–9	5048	24.5	
10–14	4803	23.3	
**Five-year period of diagnosis**			< 0.001^*^
2005–2009	8964	43.5	
2010–2014	11660	56.5	

Data represent childhood cancer cases in Iran, 2005–2014. *P*-values were calculated using the binomial proportional test for sex and diagnostic period, and the chi-square goodness-of-fit test for age group. Asterisks (*) and (**) indicate statistical significance at *P* < 0.001

### Age group-specific crude incidence rates and peak years

**Fig. 1:**
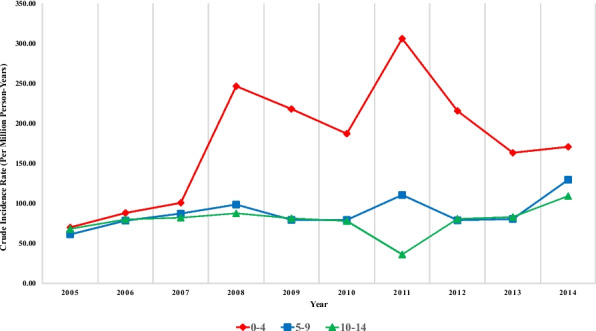
Crude incidence rate (per million person-years) of childhood cancers by age group [*Red line: ages 0–4 years; blue line: ages 5–9 years; green line: ages 10–14 years.*]

Overall, the greatest crude incidence rate (per million person-years) for childhood cancers was observed in the 0–4 year age group, although the values in the other two age groups were relatively similar (Fig. 1). The peak incidence rates for children aged 0–4, 5–9, and 10–14 years were recorded in 2011 (424.71), 2014 (131.67), and 2014 (109.30), respectively.

### Observed trends in crude and age-standardized incidence rates by sex

Over the study period, an overall increase in childhood cancer incidence was observed, although trends varied slightly by sex and age group, as shown in Table 2. Among children aged 0–4 years, the crude incidence rate rose from 147.1 (95% CI: 142.6–151.6) per million person-years in 2005–2009 to 207.6 (95% CI: 202.6–212.6) in 2010–2014, with males consistently exhibiting higher rates than females. In the 5–9 year group, incidence increased from 81.1 (95% CI: 77.8–84.5) to 96.1 (95% CI: 92.6–99.7). In contrast, the 10–14 year group showed relatively stable rates with a modest decline in both sexes, from 79.7 (95% CI: 76.6–82.8) in 2005–2009 to 77.5 (95% CI: 74.3–80.8) in 2010–2014. When all age groups were combined, the ASIR rose from 103.0 (95% CI: 100.9–105.2) in 2005–2009 to 127.8 (95% CI: 125.5–130.1) in 2010–2014, with persistently higher values among males (114.3 to 140.3) than females (91.1 to 114.7). The overall ASIR for childhood cancers in Iran was 116.2 (95% CI: 114.6–117.7) per million person-years, with corresponding values of 128.1 (95% CI: 125.8–130.5) for males and 103.6 (95% CI: 101.4–105.7) for females, confirming an upward trend and approximately 24% higher incidence in boys.

**Table 2:** Temporal trends in crude and ASIR (per million person-years) of childhood cancer by age group and sex, 2005–2014

**Age Group**	**Sex**	**Five-Year Period**		**2005–2014**
		**2005–2009**	**2010–2014**	
0–4	Male	2297 (159.6, 153.1–166.2)	3762 (229.3, 221.9–236.6)	6059 (196.7, 191.8–201.7)
	Female	1832 (133.9, 127.8–140.1)	2882 (184.9, 178.1–191.6)	4714 (161.1, 156.5–165.7)
	Total	4129 (147.1, 142.6–151.6)	6644 (207.6, 202.6–212.6)	10773 (179.4, 176.0–182.7)
5–9	Male	1363 (96.1, 91.0–101.2)	1575 (105.5, 100.3–110.7)	2938 (100.9, 97.3–104.6)
	Female	884 (65.4, 61.1–69.7)	1226 (86.3, 81.5–91.1)	2110 (76.1, 72.9–79.4)
	Total	2247 (81.1, 77.8–84.5)	2801 (96.1, 92.6–99.7)	5048 (88.8, 86.4–91.3)
10–14	Male	1432 (86.1, 81.6–90.5)	1219 (83.7, 79.0–88.3)	2651 (84.9, 81.7–88.2)
	Female	1156 (73.0, 68.8–77.2)	996 (71.1, 66.7–75.6)	2152 (72.1, 69.1–75.2)
	Total	2588 (79.7, 76.6–82.8)	2215 (77.5, 74.3–80.8)	4803 (78.7, 76.5–80.9)
ASIR	Male	114.3 (111.2–117.5)	140.3 (136.9–143.7)	128.1 (125.8–130.5)
	Female	91.1 (88.2–94.0)	114.7 (111.6–117.9)	103.6 (101.4–105.7)
	Total	103.0 (100.9–105.2)	127.8 (125.5–130.1)	116.2 (114.6–117.7)

Crude incidence rates are presented as the number of new cases per million person-years with corresponding 95% confidence intervals (CIs) for each five-year interval (2005–2009 and 2010–2014), stratified by age group and sex. Age-standardized incidence rates (ASIRs) were calculated using the new World Health Organization (WHO) world standard population

**Fig. 2:**
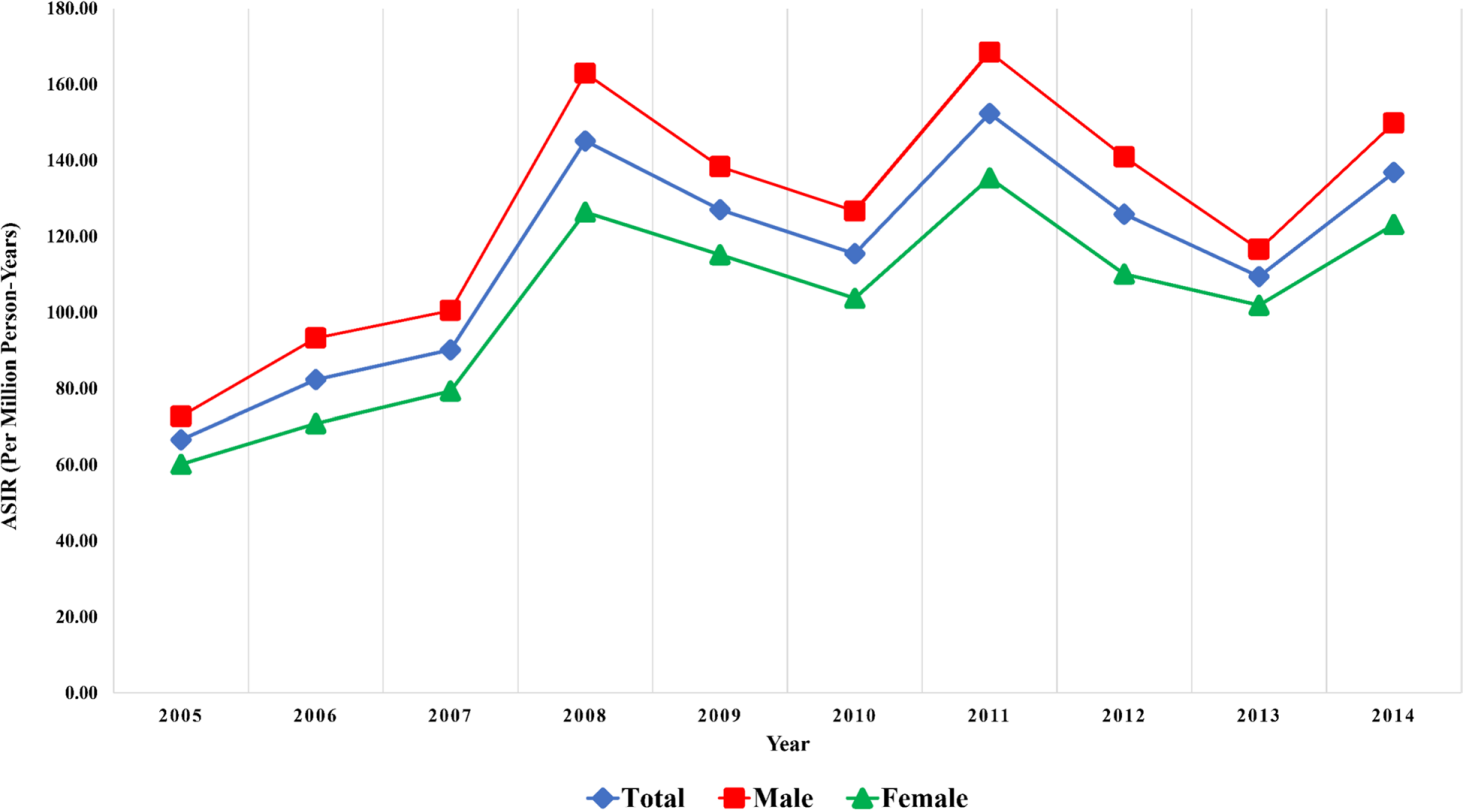
Age-standardized incidence rate (per million person-years) of childhood cancers over the 2005–2014 by sex [*Blue line: total; red line: males; green line: females.*]

The peak ASIR occurred in 2011, reaching 218.5 per million person-years in males and 170.1 in females. ASIR increased consistently across sexes, with similar trends observed (Fig. 2).

### Sex-specific ASIR by ICCC groups

Based on ICCC diagnostic classification, leukemia was the most frequent childhood cancer group in both sexes, with lymphoid leukemia accounting for the majority of cases (ASIR: 26.6 in males and 20.0 in females per million person-years), corresponding to a male-to-female (M/F) ASIR ratio of 1.3. CNS neoplasms ranked second, with an ASIR of 13.8 in males and 11.3 in females (M/F ratio: 1.2), as shown in Table 3 (Table S3 in the Supplementary File). Lymphoma and related neoplasms were the third most common group and showed a notable sex disparity, with an ASIR of 17.6 in males versus 8.5 in females, yielding the highest M/F ratio among major cancer groups (2.1). Within this group, Hodgkin lymphoma (M/F ratio: 2.0), non-Hodgkin lymphoma excluding Burkitt (M/F ratio: 1.9), and Burkitt lymphoma (M/F ratio: 3.4) demonstrated especially pronounced male predominance. Other important diagnostic groups included neuroblastoma (ASIR: 3.9 in males, 4.2 in females), soft tissue sarcoma (5.8 in males, 5.6 in females), and renal tumors (4.8 in both sexes), all with relatively balanced M/F ratios. In contrast, certain subgroups such as malignant germ cell tumors were more frequent in females (M/F ratio: 0.7), whereas carcinomas and melanomas showed higher incidence in males (ASIR: 21.8 vs. 19.8; M/F ratio: 1.3).

**Table 3:** The number of cases and ASIR (per million person-years) for childhood cancer in 2005–2014 by ICCC groups

ICCC group	Female						M/F ASIR Ratio (95% CI)	Male					
	Number of cases in age groups					ASIR per million (95% CI)		Number of cases in age groups					ASIR per million (95% CI)
	< 1	1–4	5–9	10–14	0–14	0–14	0–14	< 1	1–4	5–9	10–14	0–14	0–14
Leukaemia	107	930	813	518	2368	27.5 (26.4–28.6)	1.3 (1.2–1.4)	166	1216	1098	765	3245	35.8 (34.6–37.0)
Lymphoma & Related	101	128	197	320	746	8.5 (7.9–9.2)	2.1 (1.9–2.3)	169	231	559	651	1610	17.6 (16.8–18.5)
CNS Neoplasms	137	251	325	262	975	11.3 (10.6–12.0)	1.2 (1.1–1.3)	201	315	409	331	1256	13.8 (13.1–14.6)
Neuroblastoma	35	217	79	34	365	4.2 (3.8–4.7)	0.9 (0.8–1.1)	59	198	78	19	354	3.9 (3.5–4.3)
Retinoblastoma	22	150	20	3	195	2.3 (1.9–2.6)	1.1 (0.9–1.3)	19	170	29	3	221	2.4 (2.1–2.8)
Renal Tumours	52	226	105	30	413	4.8 (4.3–5.3)	1.0 (0.9–1.2)	65	251	94	27	437	4.8 (4.4–5.3)
Hepatic Tumours	49	51	33	18	151	1.8 (1.5–2.0)	1.4 (1.1–1.7)	94	87	21	31	233	2.6 (2.2–2.9)
Bone Tumours	16	36	116	300	468	5.3 (4.8–5.8)	1.0 (0.9–1.1)	19	52	119	284	474	5.1 (4.7–5.6)
Soft Tissue Sarcoma	51	148	112	178	489	5.6 (5.1–6.1)	1.0 (0.9–1.2)	81	157	128	165	531	5.8 (5.3–6.3)
Germ Cell Tumours	84	90	38	117	329	3.8 (3.4–4.2)	0.7 (0.6–0.8)	62	128	19	22	231	2.5 (2.2–2.9)
Other Malignant & Carcinoma & Melanoma	1266	86	129	240	1721	19.8 (18.9–20.8)	1.3 (1.3–1.4)	1548	86	167	181	1982	21.8 (20.8–22.8)
Other & Unspecified	305	176	143	132	756	8.7 (8.1–9.4)	1.4 (1.2–1.5)	437	248	217	172	1074	11.8 (11.1–12.5)
TOTAL	2225	2489	2110	2152	8976	103.6 (101.4–105.7)	1.2 (1.2–1.3)	2920	3139	2938	2651	11,648	128.1 (125.8–130.5)

The distribution of childhood cancer cases and corresponding age-standardized incidence rates (ASIRs) per million person-years among Iranian children aged 0–14 years during 2005–2014 is presented, stratified by sex, ICCC diagnostic group, and age category (< 1, 1–4, 5–9, 10–14 years). ASIRs were calculated using the direct standardization method based on the new WHO world standard population. Male-to-female ASIR ratios with 95% confidence intervals (CIs) were calculated to reflect sex-related differences in incidence. No statistical hypothesis testing was applied; all values were reported descriptively

*Abbreviations*: *ASIR* age-standardized incidence rate, *CI* confidence interval, *ICCC* International Classification of Childhood Cancer, *M/F ratio* male-to-female ASIR ratio, *WHO* World Health Organization

### Age-specific incidence by ICCC group

**Fig. 3:**
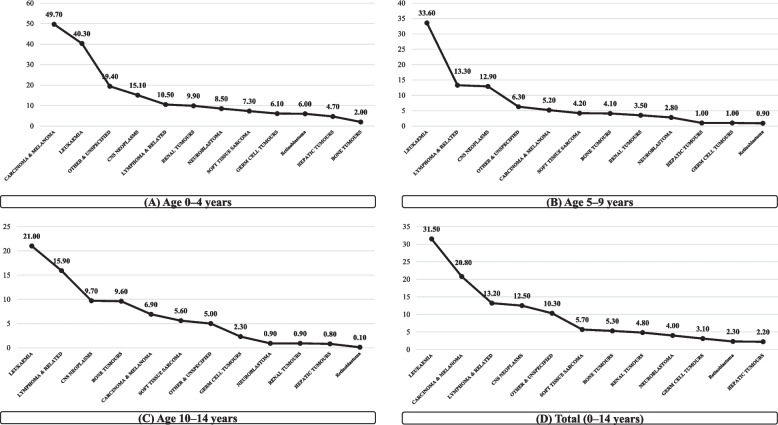
Age-specific incidence rates (per million person-years) of major childhood cancer groups. [*Panels A–C show rates in ages 0–4, 5–9, 10–14, respectively, and panel D shows all ages 0–14 for comparison*]

Table 4 (Table S4 in the Supplementary File) and Fig. 3 showed that the highest age-specific incidence rates were observed among children aged 0–4 years across most diagnostic groups, particularly for leukemia (40.3 per million), CNS neoplasms (15.1), lymphoma and related neoplasms (10.5), and renal tumors (9.9). In addition, Table 4 indicated that leukemia had the highest overall ASIR (31.7 per million person-years), with lymphoid leukemia as the predominant subtype. Lymphoma and related neoplasms ranked as the second most common group (ASIR = 13.2), showing the highest male-to-female ratio (2.1), followed by CNS neoplasms (ASIR = 12.6). Most cancer groups demonstrated increasing ASIRs between 2005–2009 and 2010–2014, with hepatic tumors showing the largest relative rise (standardized rate ratio [SRR] = 1.9), followed by carcinomas and melanomas (SRR = 1.6). In contrast, bone tumors, germ cell tumors, and retinoblastoma displayed relatively stable or declining trends (SRR ≤ 1.0). Overall, the total ASIR increased from 103.0 to 127.8 per million person-years over the study period (SRR = 1.3).

**Table 4:** Age-specific incidence rate, age-standardized incidence rates (ASIRs, per million person-years) for ICCC groups

**ICCC group**	**Age at diagnosis (age-specific rate)**				**ASIR for five-year period of diagnosis (95% CI)**		**Total ASIR**	**SRR (95% CI)**	
	**0–4**	**5–9**	**10–14**	**0–14**	**2005–2009**	**2010–2014**	**2005–2014**	**Male to female**	**2010–2014 to 2005–2009**
Leukaemia	**40.3**	**33.6**	**21.0**	**31.5**	28.5 (27.4–29.6)	34.6 (33.4–35.8)	31.7 (30.9–32.6)	**1.3 (1.2–1.4)**	1.2 (1.2–1.3)
Lymphoma & Related	**10.5**	**13.3**	**15.9**	**13.2**	13.2 (12.4–13.9)	13.1 (12.4–13.9)	13.2 (12.7–13.7)	**2.1 (1.9–2.2)**	1.0 (0.9–1.1)
CNS Neoplasms	**15.1**	**12.9**	**9.7**	**12.5**	10.2 (9.5–10.8)	14.8 (14.0–15.6)	12.6 (12.1–13.1)	**1.2 (1.1–1.3)**	1.5 (1.3–1.6)
Neuroblastoma	**8.5**	**2.8**	**0.9**	**4.0**	3.8 (3.4–4.2)	4.3 (3.9–4.7)	4.1 (3.8–4.4)	**0.9 (0.8–1.1)**	1.1 (1.0–1.3)
Retinoblastoma	**6.0**	**0.9**	**0.1**	**2.3**	2.8 (2.4–3.1)	2.0 (1.7–2.3)	2.4 (2.1–2.6)	**1.1 (0.9–1.3)**	1.0 (0.9–1.2)
Renal Tumours	**9.9**	**3.5**	**0.9**	**4.8**	4.8 (4.3–5.2)	4.9 (4.4–5.3)	4.8 (4.5–5.1)	**1.0 (0.9–1.2)**	1.0 (0.9–1.2)
Hepatic Tumours	**4.7**	**1.0**	**0.8**	**2.2**	1.5 (1.2–1.7)	2.8 (2.5–3.2)	2.2 (1.9–2.4)	**1.5 (1.2–1.8)**	1.9 (1.5–2.3)
Bone Tumours	**2.0**	**4.1**	**9.6**	**5.3**	5.2 (4.7–5.7)	5.2 (4.7–5.7)	5.2 (4.9–5.5)	**1.0 (0.9–1.1)**	1.0 (0.9–1.1)
Soft Tissue Sarcoma	**7.3**	**4.2**	**5.6**	**5.7**	5.7 (5.2–6.2)	5.7 (5.2–6.2)	5.7 (5.4–6.1)	**1.0 (0.9–1.2)**	1.0 (0.9–1.1)
Germ Cell Tumours	**6.1**	**1.0**	**2.3**	**3.1**	3.2 (2.8–3.6)	3.0 (2.7–3.4)	3.1 (2.9–3.4)	**0.7 (0.6–0.8)**	0.9 (0.8–1.1)
Other Malignant & Carcinoma & Melanoma	**49.7**	**5.2**	**6.9**	**20.8**	18.2 (17.2–19.1)	23.2 (22.3–24.2)	20.8 (20.2–21.5)	**1.1 (1.0–1.2)**	1.6 (1.6–1.7)
Other & Unspecified	**19.4**	**6.3**	**5.0**	**10.3**	6.1 (5.6–6.6)	14.1 (13.4–14.9)	10.3 (9.8–10.8)	**1.4 (1.2–1.5)**	2.3 (2.1–2.5)
**Total**	**179.4**	**88.8**	**78.7**	**115.9**	**103.0 (100.9–105.2)**	**127.8 (125.5–130.1)**	**116.2 (114.6–117.7)**	**1.2 (1.2–1.3)**	**1.3 (1.3–1.4)**

Age-specific incidence rates and age-standardized incidence rates (ASIRs) for childhood cancers among individuals aged 0–14 years were reported by ICCC group and diagnostic period (2005–2009, 2010–2014). ASIRs were calculated using the direct standardization method based on the WHO world standard population. Columns include the ASIR for each five-year period with 95% confidence intervals (CIs), the overall ASIR for 2005–2014, the male-to-female ASIR ratio, and the rate ratio (2010–2014 vs. 2005–2009) indicating changes in incidence between periods. Rate ratios and their 95% CIs were derived by dividing period-specific ASIRs, assuming a Poisson distribution for rare-event variance

*Abbreviations*: *ASIR* age-standardized incidence rate, *SRR* standardized rate ratio, *CI* confidence interval, *ICCC* International Classification of Childhood Cancer, *WHO* World Health Organization

### AAPC in childhood cancer ASIR by ICCC group

Joinpoint regression analysis of ASIRs by ICCC group revealed that most diagnostic categories showed stable trends during 2005–2014 (Table S5 in the Supplementary File). The overall ASIR for all cancers combined increased slightly but significantly, with an AAPC of 3.63% (95% CI: 0.44 to 6.65; *P* = 0.033) in females and 3.31% (95% CI: 0.20 to 6.26; *P* = 0.038) in males. Among individual ICCC groups, CNS neoplasms demonstrated a significant upward trend (AAPC = 4.91%; 95% CI: 1.27 to 8.13; *P* = 0.004), particularly astrocytoma (female: 9.95%; male: 6.10%; both *P* < 0.01). Hepatic tumors also showed a pronounced rise (AAPC = 9.41%; 95% CI: 6.09 to 12.63; *P* < 0.001), mainly driven by hepatoblastoma and hepatic carcinoma. In contrast, significant declines were observed for retinoblastoma (AAPC = –4.27%; 95% CI: –6.31 to –2.19; *P* < 0.001) and Hodgkin lymphoma (AAPC = –2.21%; 95% CI: –4.99 to –0.37; *P* = 0.022). No significant changes were detected for leukemia, bone tumors, renal tumors, soft-tissue sarcomas, or germ-cell tumors.

### Projected ASIRs to 2020

The ASIRs were projected for a six-year period (2015–2020) separately for males and females using structural time series models. According to these projections, the total ASIR of childhood cancer in Iran is estimated to reach 169.75 per million person-years in 2020 (Table S6 in the Supplementary File).

### Five-year survival by age, sex, and cancer type (2005–2014)

Of those contacted, 6,641 (≈90%) responded, while approximately 10% were unreachable despite repeated call attempts. Between 2005 and 2014, the overall five-year survival probability for childhood cancer in Iran was 81.0% (95% CI: 80.1–82.0), showing a substantial improvement from 70.2% during 2005–2009 to 81.4% in 2010–2014, as shown in Table 5. Survival varied by age group, with the highest probabilities in children diagnosed at 5–9 years (82.9%), followed by 10–14 years (82.0%), and lowest in those 0–4 years (79.6%). Females had a marginally higher five-year survival (81.3%) compared to males (80.7%). Among diagnostic groups, children with retinoblastoma (96.9%), lymphoma and related neoplasms (97.1%), soft tissue sarcoma (90.7%), and CNS neoplasms (87.6%) had the highest survival probabilities. In contrast, notably lower survival was observed in children with neuroblastoma (39.1%), hepatic tumors (66.4%), and bone tumors (65.5%). Leukemia patients demonstrated significant improvement, with survival increasing from 44.5% to 94.4% across the two periods, reaching an overall rate of 83.8%. The log-rank test indicated statistically significant differences in survival distributions by age and ICCC group (*p* < 0.05). Because cause-of-death information was not systematically verified, all survival probabilities presented here reflect overall survival.

**Table 5:** Observed 5-year survival among children with childhood cancer in Iran, 2005–2014

**Variable**	**No. of patients (deaths)**	**Five-year survival probability (95% CI)**		
		**2005–2009**	**2010–2014**	**2005–2014**
**Age**				
**0–4**	3301 (714)	67.9 (64.4–71.6)	77.3 (75.8–78.8)	79.6 (78.2–81.0) *
**5–9**	1857 (343)	67.0 (62.7–71.6)	87.8 (86.1–89.5)	82.9 (81.2–84.7)
**10–14**	1483 (287)	76.1 (72.4–79.9)	84.9 (82.7–87.2)	82.0 (80.0–83.9)
**Sex**				
Female	2846 (595)	72.6 (69.3–76.1)	80.8 (79.2–82.4)	81.3 (80.1–82.6)
Male	3795 (749)	68.5 (65.5–71.6)	81.9 (80.5–83.2)	80.7 (79.3–82.2)
**Main ICCC Groups**				
Leukaemia	2246 (374)	44.5 (40.2–49.2)	94.4 (93.4–95.5)	83.8 (82.3–85.4) *
Lymphoma & Related	804 (24)	94.9 (92.6–97.3)	98.7 (97.7–99.7)	97.1 (96.0–98.3)
CNS Neoplasms	614 (100)	37.7 (26.7–53.3)	92.3 (90.2–94.6)	87.6 (85.1–90.3)
Neuroblastoma	156 (97)	51.0 (42.0–62.1)	20.0 (12.1–33.2)	39.1 (32.1–47.6)
Retinoblastoma	224 (7)	92.5 (87.3–98.0)	100.0 (100.0–100.0)	96.9 (94.6–99.2)
Renal Tumours	348 (129)	69.8 (62.8–77.6)	58.8 (52.3–66.0)	63.5 (58.6–68.8)
Hepatic Tumours	113 (38)	0.0 (–)	72.8 (69.1–82.0)	66.4 (58.2–75.7)
Bone Tumours	287 (108)	81.5 (74.8–88.8)	54.2 (47.1–62.3)	65.5 (60.2–71.2)
Soft Tissue Sarcoma	492 (55)	96.5 (93.5–99.6)	88.3 (84.9–91.7)	90.7 (88.1–93.3)
Germ Cell Tumours	159 (43)	85.7 (72.0–100.0)	77.5 (70.9–84.8)	78.6 (72.5–85.3)
Other Malignant & Carcinoma & Melanoma	811 (270)	56.0 (39.6–79.3)	69.4 (66.2–72.7)	69.0 (65.8–72.2)
Other & Unspecified	387 (99)	96.0 (88.6–100.0)	73.0 (68.6–77.8)	74.5 (70.3–79.0)
**Total**	**6641 (1344)**	**70.2 (68.0–72.5)**	**81.4 (80.4–82.4)**	**81.0 (80.1–82.0)**

Five-year observed survival probabilities with corresponding 95% confidence intervals (CIs) are reported for childhood cancer patients in Iran across two time periods (2005–2009 and 2010–2014), as well as the entire study duration (2005–2014). Survival estimates are stratified by age group, sex, and major ICCC diagnostic categories. The column “No. of patients (deaths)” indicates the total number of patients in each subgroup, with the number of deaths shown in parentheses. Asterisks (*) denote statistically significant differences in survival distributions between variable levels based on the log-rank test (*p* < 0.05)

*Abbreviations*: *CI* confidence interval, *ICCC* International Classification of Childhood Cancer

### Provincial distribution of childhood cancer incidence

Between 2005–2009 and 2010–2014, clear temporal variations were observed in childhood cancer ASIR across Iranian provinces and between sexes (Table S7 and Figure S2 in the Supplementary File). Overall, total ASIRs increased in most provinces during 2010–2014 compared with 2005–2009, with the most substantial rises observed in Ardabil, Lorestan, Markazi, and Gilan. Ardabil showed the steepest increase, with ASIR values more than tripling over time. Sex-specific trends revealed that male ASIRs rose most markedly in Ardabil, Lorestan, and Markazi, while female ASIRs showed sharp increases in Kohgiluyeh and Boyer-Ahmad, Yazd, and Gilan. In contrast, provinces such as West Azarbaijan, East Azarbaijan, and Bushehr showed relatively stable or slightly lower ASIRs across both sexes.

## Discussion

In this nationwide study using cancer registry data, we assessed trends in childhood cancer incidence among Iranian children aged 0–14 years from 2005 to 2014 and projected crude incidence rates through 2020. After comprehensive data quality procedures, 20,624 valid cases were included. A steady increase in cancer incidence was observed across all age groups and sexes, with the highest crude rates in children aged 0–4 years. ASIRs were consistently higher in males than females, with approximately 24% greater incidence in boys. Joinpoint regression analysis showed a modest but statistically significant overall increase in ASIRs, largely driven by rising trends in central nervous system and hepatic tumors, whereas retinoblastoma and Hodgkin lymphoma declined. Leukemia, CNS neoplasms, and lymphomas were the most common cancer types in both sexes, while leukemia, lymphoma, and hepatic tumors showed the largest male-to-female ASIR disparities. The incidence of several major cancer types, including leukemia and CNS tumors, rose more sharply during the second half of the study period. Projections indicated a further increase in crude incidence rates through 2020. Additionally, five-year survival improved substantially over time, with the highest rates seen in retinoblastoma, lymphoma, and soft tissue sarcoma and the lowest in neuroblastoma, renal tumors, and bone tumors. However, survival estimates, particularly for rarer diagnostic groups, should be interpreted with caution given possible differences in follow-up completeness and case mix. These results are consistent with a recent Iranian study based on three national data sources, which also identified leukemia, lymphoma, and CNS as having the highest incidence rates among childhood cancers [25]. Furthermore, our spatial analysis revealed substantial geographical heterogeneity in childhood cancer incidence across Iranian provinces. ASIRs increased between 2005–2009 and 2010–2014 in most regions, with the sharpest rises observed in Ardabil, Lorestan, Markazi, and Gilan. Provinces such as West Azarbaijan, East Azarbaijan, and Bushehr showed relatively stable or lower ASIRs over time.

Our study estimated an overall ASIR of approximately 116 per million person-years for childhood cancers in Iran during 2005–2014, with higher rates in males (~ 128) compared to females (~ 104), likely reflecting intrinsic biological susceptibility rather than cultural factors [26–28]. To date, limited national research has addressed the ASIR of childhood cancers in Iranian populations aged 0–14 years. The most recent study by Shabani et al. reported an ASIR of 119 per million, with sex-specific rates of 134 for males and 104 for females [19]. Additionally, a literature review noted ASIRs ranging from 48 to 112 among females and 51 to 144 among males per million person-years [18]. In Europe, the ASIR for childhood cancers was approximately 176.2 per million person-years during 2014–2016 [29], while the global ASIR was estimated at 140.6 (males: 151.4; females: 129.4) based on data from 153 high-quality cancer registries across 62 countries during 2001–2010. Regional estimates showed higher ASIRs in North America, Europe, and Oceania (above 150 per million), and markedly lower rates in sub-Saharan Africa and South Asia (below 100 per million) [30]. In East Asia, ASIRs were reported at 131.9 and 116 per million in China and Japan, respectively [31, 32]. These international differences should be interpreted cautiously, as they likely reflect variations in cancer registration completeness, diagnostic capacity, and population coverage rather than true etiological disparities. Similarly, modest differences observed across Iranian provinces may relate to differences in registry infrastructure and data quality, as previously reported in national registry assessments [19, 33, 34]. Further research integrating environmental, genetic, and socioeconomic indicators is needed to clarify the underlying causes of regional variation.

We further observed an overall increasing trend in ASIRs for childhood cancers in Iran during 2005–2014, which complements the upward trend reported by Jorjani et al. for the subsequent period of 2014–2018 [35]. Similarly, Shabani et al. documented a gradual increase in incidence from 1990 to 2016, with an APC of 0.64% [19]. In comparison, our Joinpoint analysis revealed a higher AAPC of approximately 3.5% in males and females, indicating a steeper rise during the most recent decade. The upward trends were mainly driven by central nervous system and hepatic tumors, while retinoblastoma and Hodgkin lymphoma showed significant declines. Notably, the peak incidence in our study did not occur at the end of the study period, indicating a non-linear or irregular upward trend. This pattern may partly reflect improvements in diagnostic capabilities, enhanced cancer registration coverage, and reduced under reporting in earlier years [36]. In line with our results, global and European data have also demonstrated a significant positive trend in childhood cancer ASIRs over time [29]. Additionally, a longitudinal analysis from the Surveillance, Epidemiology, and End Results (SEER) program reported increasing ASIRs for pediatric cancers between 1995 and 2014 in the United States [37]. In contrast, Linabery and Ross, analyzing data from 13 American registries, reported a non significant increase across all childhood cancer types [38]. These incidence trends among children indicate key public health challenges, particularly in low- and middle-income countries like Iran, where resources supporting infrastructure and specialized pediatric oncology services may be unable to keep pace with rising cancer incidence. These trends may partly reflect improved diagnostic capacity and registry coverage over time rather than a genuine increase in underlying risk [39]. While some studies have suggested possible roles for environmental carcinogens, parental age, lifestyle factors, or inherited genetic susceptibility [40–42], the current evidence remains limited and inconsistent [43]. The etiology of most childhood cancers is still largely unclear and likely multifactorial, warranting cautious interpretation and further investigation through prospective epidemiological research [39–41, 43, 44].

Leukemia accounted for roughly one quarter of all newly diagnosed childhood cancers in our cohort, with a slightly higher contribution in males (27.9%) than females (26.4%). The overall ASIR for leukemia was about 24% higher in males than in females, consistent with international descriptive patterns. Across Western and Eastern Asia, childhood leukemia ASIRs commonly fall in the range of about 35 to 70 per million person-years and are typically higher in boys than girls, while some regions in Western and Middle Africa and parts of South-Central Asia report markedly lower rates, a pattern that is plausibly influenced by diagnostic limitations and incomplete registration [45, 46]. These observations align with the notion that both biological differences and system-level factors such as registry completeness and access to pediatric oncology shape observed incidence.

Lymphoid leukemia was the predominant subtype, with ASIRs of approximately 27 and 20 per million person-years for males and females, respectively, and it was the only subtype that rose significantly in the second half of the study period. Countries with higher HDI values tend to report higher leukemia ASIRs, and several high-income settings in North America and Western Europe have documented comparatively elevated lymphoid leukemia rates, which may reflect more comprehensive registration and diagnostic capacity [45, 47–49]. By contrast, lower-income Asian settings often report lower incidence, likely due in part to underdiagnosis and incomplete registry data capture [50]. Taken together, our subtype-specific findings are consistent with these global patterns and indicate that recent increases in childhood leukemia in Iran are largely driven by lymphoid disease.

In our registry, AML occurred far less often than lymphoid leukemia and showed no material change across the two five-year periods, with only a modest male excess. This pattern, including a small contribution of AML to the childhood leukemia burden, slight male predominance, and broadly stable temporal behavior, is consistent with external evidence. Global Burden of Disease estimates (GBD 2023) for ages 0–14 report AML incidence rates of about 0.91 per 100,000 in 2005 declining to about 0.75 in 2014 worldwide, and about 1.23 in 2005 declining to about 1.06 in 2014 in North Africa and the Middle East, which supports relative rarity and limited secular change in this age range [51]. Our ASIRs are slightly higher than these GBD rates; this likely reflects methodological differences (e.g., standard population/definitions) and small-number variability in a rare outcome, rather than a true underlying excess.

The present study revealed a notable improvement in five-year survival among Iranian children with cancer over the study period, with overall survival approaching 80%. Survival was generally lower among younger children, particularly those under five years old, compared to older age groups. Among diagnostic categories, neuroblastoma demonstrated the poorest prognosis, while retinoblastoma, lymphoma, and soft tissue sarcoma were associated with the most favorable outcomes. These findings align with a national study by Keramatinia et al., which identified neuroblastoma, brain tumors, and sarcomas as having significantly higher mortality risks than leukemia, with hazard ratios exceeding 2.0. Additionally, that study found older age to be a significant predictor of survival [52]. Our overall survival estimate is also comparable to the global five-year survival of approximately 78% reported by Miller et al., suggesting that survival outcomes in Iran have improved, although they continue to vary by cancer type and patient characteristics [37]. These variations in survival can be attributed to a range of interrelated factors, including the timing of diagnosis, cancer subtype, treatment modality, and access to specialized pediatric oncology care. Both national and international studies have highlighted that multidisciplinary treatment approaches, higher socioeconomic status, and robust healthcare infrastructure are associated with better survival outcomes [25, 53, 54]. In contrast, diagnostic delays, insufficient access to pediatric oncology services, and inadequate follow-up care contribute to poorer long-term outcomes [55–57].

The reported five-year survival of approximately 90% for pediatric soft-tissue sarcomas should be interpreted with caution. Despite the registry’s robust follow-up and data validation system, a degree of selection bias may still exist in this diagnostic group. In particular, children with advanced or relapsed disease who generally have poorer prognoses may have been more likely to experience incomplete follow-up or non-response during post-treatment verification, resulting in a modest upward bias in observed survival. Similar phenomena have been described in population-based cancer registries where incomplete ascertainment of fatal outcomes can inflate survival estimates, especially in heterogeneous diagnostic groups [58–60]. Furthermore, the favorable case mix in this cohort, dominated by localized, low-grade, non-rhabdomyosarcoma soft-tissue sarcomas, could also contribute to higher survival compared with mixed or hospital-based cohorts. Importantly, since cause-of-death data were not uniformly available, all survival analyses in this study represent *overall survival* rather than cancer-specific survival. Collectively, these considerations highlight the need for cautious interpretation of survival data in registry-based analyses, particularly for rare pediatric malignancies such as soft-tissue sarcomas.

Our findings align with and extend previous Iranian spatiotemporal studies. Similar to the findings of Saffar et al. (2005–2013) [61], we observed increasing ASIRs over time and clear geographic disparities, with higher rates in central and northern provinces and lower rates in the northwest and southeast. Hashemi et al. (1999–2016) also identified elevated relative risks in several provinces using Bayesian spatiotemporal models, consistent with our results showing rising trends in Ardabil, Lorestan, Markazi, and Gilan and relative stability in West and East Azerbaijan and Bushehr [33]. Likewise, our sex-specific findings mirror those of Saffar et al. and Jorjani et al. (2014–2018), which reported higher incidence in boys and high-risk clusters in central Iran, including Isfahan, Yazd, and Tehran [35, 61]. Despite differences in study periods and modeling approaches, all evidence points to a consistent national pattern of increasing incidence, male predominance, and persistent spatial clustering of childhood cancers in Iran.

In the present study, future patterns of childhood cancer incidence were projected through 2020 using structural time series models fitted to the WHO-standardized ASIRs. This modeling approach decomposes the observed series into stochastic trend and irregular components, allowing for robust estimation of long-term changes while minimizing the influence of short-term fluctuations. Because nationwide registry data after 2014 were incomplete and inconsistently reported, the projection was extended through 2020 to make optimal use of the available information and to provide a plausible continuation of the observed trend. These short-term projections were intended to contextualize how incidence rates may have evolved during years with limited data coverage, rather than to produce long-range policy forecasts. The model assumes a smooth and continuous temporal trajectory without abrupt structural breaks, stable registry coding and ascertainment practices relative to the 2005–2014 period, and independent, homoscedastic residuals. The moderate upward trajectory of projected ASIRs, reaching 169.8 per million person-years by 2020, likely reflects multiple factors, including gradual improvements in cancer registration coverage, enhanced diagnostic facilities, and increased awareness leading to earlier case detection, rather than a true surge in underlying risk [62–65]. Consistent with this interpretation, sex-specific projections showed slightly higher rates among males, in line with the historical male predominance observed in most pediatric cancer registries [19]. Together, these results suggest that the expected rise in incidence primarily mirrors progress in case ascertainment and demographic stabilization rather than an epidemiological escalation of childhood cancer burden.

In this study, ASIRs were calculated using the WHO world standard population (2000–2025), representing a modern approximation of worldwide age distributions and better accommodating demographic shifts in developed and developing countries. With reference to Segi's 1960 s standard, which assigns greater contribution to younger ages, the WHO standard minimizes bias towards early-life occurrences and produces estimates that are more appropriate for current-era cancer epidemiology and policymaking [21, 66]. Robson et al. emphasized that the selection of a standard population may significantly impact absolute rates, rate ratios, and even rankings of diseases themselves and that the utilization of a demographically current reference is critical for such purposes [67]. Likewise, Wyper et al. demonstrated that differences between Segi and WHO populations can markedly affect burden-of-disease metrics such as DALYs, supporting the use of WHO weights for modern analyses [68]. Although IARC and GLOBOCAN continue to employ Segi’s standard for historical comparability, numerous recent registry-based and national cancer studies in Iran have adopted the WHO standard, promoting internal consistency and interpretability across time [69–78]. For transparency, it should be noted that absolute ASIR values may differ slightly if recalculated with Segi’s population, though temporal and inter-group trends remain directionally consistent.

This study has several limitations that should be acknowledged. First, although Joinpoint regression was applied to identify potential changes in temporal trends, the relatively short 10-year observation window (2005–2014) limited the number of possible joinpoints and reduced statistical power to detect subtle inflection points. Consequently, some AAPC and APC estimates had wide CIs, reflecting uncertainty driven by the small number of data points available for model fitting [79, 80]. Second, a relatively high proportion of cancers were categorized as "unspecified," suggesting potential limitations in diagnostic precision or registry completeness. Third, although ASIRs are widely used to allow international comparisons, they do not reflect the absolute number of cases and are inherently dependent on the choice of the standard population, which may introduce some degree of bias. Fourth, due to the absence of data on potential etiological risk factors in the Iran National Cancer Registry, we were unable to investigate the role of environmental exposures, genetic predispositions, or socioeconomic determinants in shaping the observed cancer incidence patterns. Fifth, although survival data were gathered via follow-up telephone interviews, approximately 10% of the sampled individuals could not be reached despite repeated contact attempts, introducing the potential for loss to follow-up. However, this risk was minimized through a random sampling design, a high overall response rate (~ 90%), and consistency of demographic and diagnostic distributions between respondents and the total registry population, suggesting limited bias in survival estimates. In addition, recall bias and reporting inaccuracies may have occurred, however, these risks were mitigated through cross-validation of key information and structured data-collection procedures. Finally, underdiagnosis and incomplete registry coverage may still exist in earlier years of the study, potentially leading to an underestimation of incidence rates, despite rigorous quality control efforts such as the exclusion of non-Iranian and duplicate cases, and validation of tumor–sex, tumor–age, and morphology–topography consistency.

Despite these limitations, the study has several notable strengths. It is the first nationwide analysis in Iran to comprehensively estimate ASIRs for all 12 ICCC main groups and 45 subgroups of childhood cancer, providing a detailed epidemiological profile that was previously lacking. Another major strength lies in the extended observation period (2005 to 2020), which allows for both historical trend analysis and future projections. Additionally, the large registry-based sample, comprising more than 20,000 validated cases from all provinces, ensures a high degree of representativeness for Iranian children aged 0 to 14 years. The use of standardized data cleaning protocols and WHO standard population weights further enhances the reliability and comparability of the incidence estimates. Moreover, the availability of follow-up data for a large subset of registry cases enabled estimation of five-year survival probabilities, providing the first national pediatric cancer survival estimates in Iran. Taken together, these strengths support the robustness of the findings and offer valuable evidence regarding the epidemiology of childhood cancer in Iran.

## Conclusion

This nationwide analysis of more than 20,000 childhood cancer cases provides the most comprehensive evidence to date on incidence and survival patterns in Iran. Between 2005 and 2014, both crude and age-standardized incidence rates increased steadily, with higher rates in boys and in the youngest age group (0–4 years). Leukemia, CNS neoplasms, and lymphomas were the predominant cancer types, and Joinpoint analysis indicated modest but significant upward trends, particularly for CNS and hepatic tumors. Although survival outcomes appeared to improve over time, these estimates should be interpreted cautiously given possible differences in follow-up completeness and case mix, and notable variations persisted across cancer types and age groups. These findings underscore measurable progress in pediatric oncology outcomes while highlighting the need to sustain and expand national childhood-cancer surveillance, diagnostic capacity, and equitable access to specialized care. Strengthening registry completeness, early detection, and multidisciplinary treatment pathways can help ensure that recent gains translate into continued improvements in survival and quality of care for Iranian children with cancer.

## Supplementary Information

Supplementary Material 1

## Data Availability

The data used in this study were obtained from the Iranian National Cancer Registry system under official permission. Due to data confidentiality policies, the full dataset is not publicly available. However, a sample of the data may be made available upon reasonable request to the corresponding author.
