# Supplementary material for: Nationwide trends in childhood cancer incidence and survival in Iran: analysis of national cancer registry data, 2005–2014
Source: BMC Pediatr. 2025 Dec 5;25:977. doi: 10.1186/s12887-025-06363-4 (PMC12679737; doi:10.1186/s12887-025-06363-4)
Supplement: Supplementary file 1 — Supplementary Material 1 [file 12887_2025_6363_MOESM1_ESM.docx]

**Nationwide Trends in Childhood Cancer Incidence and Survival in Iran: Analysis of National Cancer Registry Data, 2005–2014**

Table S1. Structured Follow-up Verification Checklist Used in Telephone Interviews

| **Domain** | **Item** | **Response Type** | **Notes** |
| --- | --- | --- | --- |
| **Identity verification** | Patient’s full name (confirm from registry) | Yes/No | If no → verify with caregiver or other family member |
|  | Patient’s sex (confirm from registry) | Yes/No | No new information is requested unless discrepancy exists |
|  | Date of birth or age at diagnosis (confirm from registry) | Yes/No | Registry data are retained if discrepancies cannot be resolved |
|  | Province of residence at diagnosis | Confirm / Correct if needed | Used if mismatch is identified |
| **Diagnosis verification** | Confirmation of primary cancer diagnosis as reported in registry | Confirm / Not sure / Incorrect | Registry diagnosis remains the reference if unclear |
| **Vital status** | Current vital status | Alive / Deceased | If deceased → proceed to next item |
|  | Date of death (exact or approximate) | Numeric / Month–Year | If unavailable, approximate date is recorded |
|  | Date of last confirmed contact (for living patients) | Numeric / Month–Year | Documented based on phone interview date if no earlier information is available |
| **Voluntary information** | Cause of death (if offered voluntarily) | Free text | Optional field; not used for analysis due to verification limitations |
| **Interview process** | Consent from parent/legal guardian or next-of-kin obtained | Yes/No | Required before interview continues |
|  | Interview completed successfully | Yes/No | If No → record reason (e.g., refused, disconnected) |

Table S2. Annual Duplicate Removal Summary from the Childhood Cancer Registry, 2005–2014

| **Year** | **Before Duplicate** **Removal** | **After Duplicated Removal** | **Percentage of Exclusion** |
| --- | --- | --- | --- |
| 2005 | 1201 | 1198 | 0.25 |
| 2006 | 1476 | 1472 | 0.27 |
| 2007 | 1676 | 1591 | 5.07 |
| 2008 | 2874 | 2815 | 2.05 |
| 2009 | 2660 | 2598 | 2.33 |
| 2010 | 2666 | 2633 | 1.24 |
| 2011 | 3865 | 3414 | 11.67 |
| 2012 | 3072 | 2658 | 13.48 |
| 2013 | 2683 | 2227 | 17.00 |
| 2014 | 2716 | 2596 | 4.42 |
| Total | 24889 | 23202 | 6.78 |

Note: The number of extracted records was assessed before and after duplicate removal. The percentage indicates the proportion of duplicates excluded annually. The higher exclusion percentages in 2011–2013 reflect duplicate submissions during the registry’s transition to semi-electronic reporting and do not indicate data loss or under-ascertainment.

Table S3. The number of cases and ASIR (per million person-years) for childhood cancer in 2005–2014 by ICCC group and subgroups

| **ICCC group** | **Female** | | | | | | **M/F**  **ASIR**  **Ratio**  **(95% CI)** | **Male** | | | | | |
| --- | --- | --- | --- | --- | --- | --- | --- | --- | --- | --- | --- | --- | --- |
|  | **Number of cases in age groups** | | | | | **ASIR Per Million**  **(95% CI)** |  | **Number of cases in age groups** | | | | | **ASIR Per Million**  **(95% CI)** |
|  | **<1** | **1-4** | **5-9** | **10-14** | **0-14** | **0-14** | **0-14** | **<1** | **1-4** | **5-9** | **10-14** | **0-14** | **0-14** |
| **LEUKAEMIA** | **107** | **930** | **813** | **518** | **2368** | **27.5 (26.4-28.6)** | **1.3 (1.2-1.4)** | **166** | **1216** | **1098** | **765** | **3245** | **35.8 (34.6-37.0)** |
| Lymphoid | 45 | 709 | 640 | 330 | 1724 | 20.0 (19.1-21.0) | 1.3 (1.2-1.4) | 83 | 950 | 853 | 518 | 2404 | 26.6 (25.5-27.6) |
| Acute myeloid | 24 | 89 | 87 | 112 | 312 | 3.6 (3.2-4.0) | 1.2 (1.1-1.4) | 27 | 122 | 119 | 137 | 405 | 4.4 (4.0-4.9) |
| CMD | 9 | 12 | 10 | 24 | 55 | 0.6 (0.5-0.8) | 0.9 (0.6-1.3) | 13 | 9 | 10 | 19 | 51 | 0.6 (0.4-0.7) |
| MDS and other | 3 | 3 |  | 1 | 7 | 0.1 (0.0-0.1) | 2.5 (1.1-5.7) | 3 | 10 | 2 | 3 | 18 | 0.2 (0.1-0.3) |
| Unspecified and other specified | 26 | 117 | 76 | 51 | 270 | 3.1 (2.8-3.5) | 1.3 (1.1-1.5) | 40 | 125 | 114 | 88 | 367 | 4.0 (3.6-4.5) |
| **LYMPHOMA & RELATED** | **101** | **128** | **197** | **320** | **746** | **8.5 (7.9-9.2)** | **2.1 (1.9-2.3)** | **169** | **231** | **559** | **651** | **1610** | **17.6 (16.8-18.5)** |
| Hodgkin | 15 | 22 | 78 | 184 | 299 | 3.4 (3.0-3.8) | 2.0 (1.7-2.3) | 39 | 59 | 226 | 297 | 621 | 6.8 (6.3-7.3) |
| Non-Hodgkin except BL | 66 | 57 | 64 | 86 | 273 | 3.1 (2.8-3.5) | 1.9 (1.7-2.2) | 91 | 93 | 157 | 212 | 553 | 6.1 (5.5-6.6) |
| Burkitt (BL) | 1 | 21 | 25 | 13 | 60 | 0.7 (0.5-0.9) | 3.4 (2.6-4.4) | 6 | 40 | 103 | 63 | 212 | 2.3 (2.0-2.7) |
| lymphoreticular | 3 | 7 | 2 | 2 | 14 | 0.2 (0.1-0.2) | 1.3 (0.7-2.6) | 5 | 9 | 4 | 1 | 19 | 0.2 (0.1-0.3) |
| Unspecified | 16 | 21 | 28 | 35 | 100 | 1.1 (0.9-1.4) | 2.0 (1.5-2.5) | 28 | 30 | 69 | 78 | 205 | 2.2 (1.9-2.6) |
| **CNS NEOPLASMS** | **137** | **251** | **325** | **262** | **975** | **11.3 (10.6-12.0)** | **1.2 (1.1-1.3)** | **201** | **315** | **409** | **331** | **1256** | **13.8 (13.1-14.6)** |
| Ependymoma and choroid plexus tumor | 5 | 52 | 32 | 24 | 113 | 1.3 (1.1-1.5) | 1.2 (0.9-1.5) | 14 | 61 | 36 | 30 | 141 | 1.6 (1.3-1.8) |
| Astrocytoma | 67 | 63 | 103 | 110 | 343 | 4.0 (3.5-4.4) | 1.1 (1.0-1.3) | 97 | 63 | 125 | 118 | 403 | 4.4 (4.0-4.9) |
| Intracranial and intraspinal embryonal tumors | 14 | 60 | 87 | 65 | 226 | 2.6 (2.3-3.0) | 1.5 (1.2-1.7) | 21 | 100 | 135 | 92 | 348 | 3.8 (3.4-4.2) |
| Other gliomas | 14 | 7 | 24 | 20 | 65 | 0.8 (0.6-0.9) | 1.3 (0.9-1.8) | 22 | 20 | 22 | 25 | 89 | 1.0 (0.8-1.2) |
| Other specified | 3 | 2 | 6 | 3 | 14 | 0.2 (0.1-0.2) | 1.4 (0.7-2.7) | 5 | 2 | 7 | 6 | 20 | 0.2 (0.1-0.3) |
| Unspecified CNS | 34 | 67 | 73 | 40 | 214 | 2.5 (2.2-2.8) | 1.1 (0.9-1.4) | 42 | 69 | 84 | 60 | 255 | 2.8 (2.5-3.2) |
| **NEUROBLASTOMA** | **35** | **217** | **79** | **34** | **365** | **4.2 (3.8-4.7)** | **0.9 (0.8-1.1)** | **59** | **198** | **78** | **19** | **354** | **3.9 (3.5-4.3)** |
| Neuroblastoma and ganglioneuroblastoma | 33 | 215 | 78 | 31 | 357 | 4.1 (3.7-4.6) | 0.9 (0.8-1.1) | 58 | 196 | 77 | 18 | 349 | 3.9 (3.5-4.3) |
| peripheral nervous | 2 | 2 | 1 | 3 | 8 | 0.1 (0.0-0.2) | 0.6 (0.2-1.8) | 1 | 2 | 1 | 1 | 5 | 0.1 (0.0-0.1) |
| **RETINOBLASTOMA** | **22** | **150** | **20** | **3** | **195** | **2.3 (1.9-2.6)** | **1.1 (0.9-1.3)** | **19** | **170** | **29** | **3** | **221** | **2.4 (2.1-2.8)** |
| **RENAL TUMOURS** | **52** | **226** | **105** | **30** | **413** | **4.8 (4.3-5.3)** | 1.0 (0.9-1.2) | **65** | **251** | **94** | **27** | **437** | **4.8 (4.4-5.3)** |
| Nephroblastoma | 22 | 207 | 92 | 21 | 342 | 4.0 (3.6-4.4) | 0.9 (0.8-1.1) | 22 | 220 | 81 | 18 | 341 | 3.8 (3.4-4.2) |
| Renal carcinomas | 26 | 9 | 8 | 9 | 52 | 0.6 (0.4-0.8) | 1.1 (0.7-1.5) | 39 | 11 | 4 | 4 | 58 | 0.6 (0.5-0.8) |
| Unspecified | 4 | 10 | 5 |  | 19 | 0.2 (0.1-0.3) | 1.9 (1.1-3.2) | 4 | 20 | 9 | 5 | 38 | 0.4 (0.3-0.6) |
| **HEPATIC TUMOURS** | **49** | **51** | **33** | **18** | **151** | **1.8 (1.5-2.0)** | **1.4 (1.1-1.7)** | **94** | **87** | **21** | **31** | **233** | **2.6 (2.2-2.9)** |
| Hepatoblastoma | 14 | 38 | 15 | 3 | 70 | 0.8 (0.6-1.0) | 1.4 (1.0-1.9) | 21 | 73 | 3 | 10 | 107 | 1.2 (1.0-1.4) |
| Hepatic carcinomas | 17 | 6 | 10 | 9 | 42 | 0.5 (0.3-0.6) | 1.4 (0.9-2.1) | 38 | 2 | 7 | 15 | 62 | 0.7 (0.5-0.8) |
| Unspecified | 18 | 7 | 8 | 6 | 39 | 0.5 (0.3-0.6) | 1.6 (1.1-2.3) | 35 | 12 | 11 | 6 | 64 | 0.7 (0.5-0.9) |

Table S3 (Continue). The number of cases and ASIR (per million person-years) for childhood cancer in 2005–2014 by ICCC group and subgroups

| **ICCC group** | **Female** | | | | | | **M/F**  **ASIR**  **Ratio**  **(95% CI)** | **Male** | | | | | |
| --- | --- | --- | --- | --- | --- | --- | --- | --- | --- | --- | --- | --- | --- |
|  | **Number of cases in age groups** | | | | | **ASIR Per Million (95% CI)** |  | **Number of cases in age groups** | | | | | **ASIR Per Million (95% CI)** |
|  | **<1** | **1-4** | **5-9** | **10-14** | **0-14** | **0-14** | **0-14** | **<1** | **1-4** | **5-9** | **10-14** | **0-14** | **0-14** |
| **BONE TUMOURS** | **16** | **36** | **116** | **300** | **468** | **5.3 (4.8-5.8)** | **1.0 (0.9-1.1)** | **19** | **52** | **119** | **284** | **474** | **5.1 (4.7-5.6)** |
| Osteosarcomas | 3 | 7 | 50 | 189 | 249 | 2.8 (2.5-3.1) | 0.9 (0.7-1.0) | 3 | 7 | 52 | 165 | 227 | 2.4 (2.1-2.8) |
| Chondrosarcomas | 4 |  | 3 | 6 | 13 | 0.1 (0.1-0.2) | 1.1 (0.5-2.3) | 5 | 2 | 1 | 7 | 15 | 0.2 (0.1-0.2) |
| Ewing and related | 1 | 12 | 51 | 75 | 139 | 1.6 (1.3-1.9) | 0.9 (0.7-1.2) | 3 | 24 | 41 | 65 | 133 | 1.5 (1.2-1.7) |
| Other specified | 1 | 1 | 4 | 7 | 13 | 0.1 (0.1-0.2) | 1.5 (0.7-3.0) | 2 | 2 | 8 | 8 | 20 | 0.2 (0.1-0.3) |
| Unspecified | 7 | 16 | 8 | 23 | 54 | 0.6 (0.5-0.8) | 1.4 (1.0-2.0) | 6 | 17 | 17 | 39 | 79 | 0.9 (0.7-1.0) |
| **SOFT TISSUE SARCOMA** | **51** | **148** | **112** | **178** | **489** | **5.6 (5.1-6.1)** | **1.0 (0.9-1.2)** | **81** | **157** | **128** | **165** | **531** | **5.8 (5.3-6.3)** |
| Rhabdomyosarcomas | 4 | 82 | 57 | 44 | 187 | 2.2 (1.9-2.5) | 1.0 (0.8-1.3) | 12 | 94 | 57 | 39 | 202 | 2.2 (1.9-2.5) |
| Fibrosarcomas and other | 11 | 7 | 6 | 15 | 39 | 0.4 (0.3-0.6) | 1.1 (0.7-1.7) | 14 | 9 | 8 | 14 | 45 | 0.5 (0.3-0.6) |
| Kaposi sarcoma | 5 |  | 1 | 1 | 7 | 0.1 (0.0-0.1) | 1.1 (0.4-3.0) | 6 |  | 1 | 1 | 8 | 0.1 (0.0-0.1) |
| Other specified | 24 | 42 | 28 | 76 | 170 | 1.9 (1.6-2.2) | 1.0 (0.8-1.3) | 37 | 33 | 41 | 72 | 183 | 2.0 (1.7-2.3) |
| Unspecified | 7 | 17 | 20 | 42 | 86 | 1.0 (0.8-1.2) | 1.0 (0.8-1.4) | 12 | 21 | 21 | 39 | 93 | 1.0 (0.8-1.2) |
| **GERM CELL TUMOURS** | **84** | **90** | **38** | **117** | **329** | **3.8 (3.4-4.2)** | **0.7 (0.6-0.8)** | **62** | **128** | **19** | **22** | **231** | **2.5 (2.2-2.9)** |
| Intracranial and intraspinal | 2 | 5 | 3 | 9 | 19 | 0.2 (0.1-0.3) | 0.6 (0.3-1.2) | 3 | 1 | 2 | 6 | 12 | 0.1 (0.1-0.2) |
| Malignant extracranial and extragonadal | 21 | 61 | 9 | 16 | 107 | 1.2 (1.0-1.5) | 0.4 (0.3-0.6) | 8 | 30 | 3 | 5 | 46 | 0.5 (0.4-0.7) |
| Malignant gonadal germ cell | 10 | 20 | 17 | 80 | 127 | 1.4 (1.2-1.7) | 1.2 (0.9-1.5) | 44 | 90 | 9 | 9 | 152 | 1.7 (1.4-1.9) |
| Gonadal carcinomas | 32 | 1 | 3 | 7 | 43 | 0.5 (0.3-0.6) | 0.0 (0.0-0.1) | 2 |  |  |  | 2 | 0.0 (0.0-0.1) |
| Other and unspecified gonadal | 19 | 3 | 6 | 5 | 33 | 0.4 (0.3-0.5) | 0.6 (0.3-1.0) | 5 | 7 | 5 | 2 | 19 | 0.2 (0.1-0.3) |
| **OTHER MALIGNANT & CARCINOMA & MELANOMA** | **1266** | **86** | **129** | **240** | **1721** | **19.8 (18.9-20.8)** | **1.3 (1.3-1.4)** | **1548** | **86** | **167** | **181** | **1982** | **21.8 (20.8-22.8)** |
| Adrenocortical | 1 | 5 |  | 2 | 8 | 0.1 (0.0-0.2) | 0.2 (0.1-1.0) |  | 1 | 1 |  | 2 | 0.0 (0.0-0.1) |
| Thyroid | 83 | 7 | 7 | 106 | 203 | 2.3 (2.0-2.6) | 0.3 (0.3-0.4) | 18 | 6 | 9 | 40 | 73 | 0.8 (0.6-1.0) |
| Nasopharyngeal | 2 | 1 | 4 | 5 | 12 | 0.1 (0.1-0.2) | 1.7 (0.9-3.4) | 4 |  | 2 | 16 | 22 | 0.2 (0.1-0.3) |
| Malignant melanomas | 21 | 5 | 5 | 8 | 39 | 0.4 (0.3-0.6) | 1.1 (0.7-1.6) | 33 | 4 | 2 | 4 | 43 | 0.5 (0.3-0.6) |
| Skin carcinomas |  | 11 | 30 | 34 | 75 | 0.9 (0.7-1.1) | 1.4 (1.0-1.9) |  | 27 | 46 | 36 | 109 | 1.2 (1.0-1.4) |
| Other & unspecified | 1159 | 57 | 83 | 85 | 1384 | 16.0 (15.2-16.9) | 1.2 (1.1-1.3) | 1493 | 48 | 107 | 85 | 1733 | 19.1 (18.2-20.0) |
| **OTHER & UNSPECIFIED** | **305** | **176** | **143** | **132** | **756** | **8.7 (8.1-9.4)** | **1.4 (1.2-1.5)** | **437** | **248** | **217** | **172** | **1074** | **11.8 (11.1-12.5)** |
| Other specified | 3 | 3 | 2 |  | 8 | 0.1 (0.0-0.2) | 1.9 (0.8-4.3) | 4 | 10 | 1 | 1 | 16 | 0.2 (0.1-0.3) |
| Other unspecified | 302 | 173 | 141 | 132 | 748 | 8.6 (8.0-9.3) | 1.3 (1.2-1.5) | 433 | 238 | 216 | 171 | 1058 | 11.7 (10.9-12.4) |
| **TOTAL** | **2225** | **2489** | **2110** | **2152** | **8976** | **103.6 (101.4-105.7)** | **1.2 (1.2-1.3)** | **2920** | **3139** | **2938** | **1651** | **11648** | **128.1 (125.8-130.5)** |

Table S4. Age-specific incidence rate, ASIRs (per million person-years) for ICCC groups and subgroups

| **ICCC group** | **Age at Diagnosis (Age-Specific Rate)** | | | | **ASIR for Five-year period of diagnosis (95% CI)** | | **Total ASIR** | **SRR (95% CI)** | |
| --- | --- | --- | --- | --- | --- | --- | --- | --- | --- |
|  | **0-4** | **5-9** | **10-14** | **0-14** | **2005-2009** | **2010-2014** | **2005-2014** | **Male to Female** | **2010-2014 to 2005-2009** |
| **LEUKAEMIA** | **40.3** | **33.6** | **21.0** | **31.5** | **28.5 (27.4-29.6)** | **34.6 (33.4-35.8)** | **31.7 (30.9-32.6)** | **1.3 (1.2-1.4)** | 1.2 (1.2-1.3) |
| Lymphoid | 29.8 | 26.3 | 13.9 | 23.2 | 21.7 (20.7-22.7) | 24.9 (23.8-25.9) | 23.5 (22.8-24.2) | 1.3 (1.2-1.4) | 1.1 (1.1-1.2) |
| Acute myeloid | 4.4 | 3.6 | 4.1 | 4.0 | 4.1 (3.7-4.5) | 3.9 (3.5-4.3) | 4.0 (3.7-4.3) | 1.2 (1.1-1.4) | 1.0 (0.8-1.1) |
| CMD | 0.7 | 0.4 | 0.7 | 0.6 | 0.6 (0.5-0.8) | 0.6 (0.4-0.7) | 0.6 (0.5-0.7) | 0.9 (0.6-1.3) | 0.9 (0.6-1.2) |
| MDS and other | 0.3 | 0.0 | 0.1 | 0.1 | 0.1 (0.0-0.2) | 0.2 (0.1-0.3) | 0.1 (0.1-0.2) | 2.5 (1.1-5.7) | 1.9 (0.9-4.4) |
| Unspecified and other specified | 5.1 | 3.3 | 2.3 | 3.6 | 2.0 (1.7-2.3) | 5.1 (4.6-5.5) | 3.6 (3.3-3.9) | 1.3 (1.1-1.5) | 2.5 (2.1-3.0) |
| **LYMPHOMA & RELATED** | **10.5** | **13.3** | **15.9** | **13.2** | **13.2 (12.4-13.9)** | **13.1 (12.4-13.9)** | **13.2 (12.7-13.7)** | **2.1 (1.9-2.3)** | 1.0 (0.9-1.1) |
| Hodgkin | 2.2 | 5.3 | 7.9 | 5.2 | 5.6 (5.1-6.1) | 4.6 (4.2-5.1) | 5.1 (4.8-5.5) | 2.0 (1.7-2.3) | 0.8 (0.7-0.9) |
| Non-Hodgkin except BL | 5.1 | 3.9 | 4.9 | 4.6 | 4.2 (3.8-4.6) | 5.0 (4.5-5.4) | 4.6 (4.3-4.9) | 1.9 (1.7-2.2) | 1.2 (1.0-1.4) |
| Burkitt (BL) | 1.1 | 2.3 | 1.2 | 1.5 | 1.6 (1.3-1.9) | 1.5 (1.2-1.7) | 1.5 (1.4-1.7) | 3.4 (2.6-4.4) | 0.9 (0.7-1.1) |
| lymphoreticular | 0.4 | 0.1 | 0.0 | 0.2 | 0.2 (0.1-0.2) | 0.2 (0.1-0.3) | 0.2 (0.1-0.3) | 1.3 (0.7-2.6) | 1.4 (0.7-2.8) |
| Unspecified | 1.6 | 1.7 | 1.9 | 1.7 | 1.6 (1.3-1.8) | 1.8 (1.5-2.1) | 1.7 (1.5-1.9) | 2.0 (1.5-2.5) | 1.1 (0.9-1.4) |
| **CNS NEOPLASMS** | **15.1** | **12.9** | **9.7** | **12.5** | **10.2 (9.5-10.8)** | **14.8 (14.0-15.6)** | **12.6 (12.1-13.1)** | **1.2 (1.1-1.3)** | 1.5 (1.3-1.6) |
| Ependymoma and choroid plexus tumor | 2.2 | 1.2 | 0.9 | 1.4 | 1.3 (1.0-1.5) | 1.6 (1.3-1.8) | 1.4 (1.3-1.6) | 1.2 (0.9-1.5) | 1.3 (1.0-1.6) |
| Astrocytoma | 4.8 | 4.0 | 3.7 | 4.2 | 3.9 (3.5-4.3) | 4.4 (4.0-4.9) | 4.2 (3.9-4.5) | 1.1 (1.0-1.3) | 1.1 (1.0-1.3) |
| Intracranial and intraspinal embryonal tumors | 3.2 | 3.9 | 2.6 | 3.2 | 3.2 (2.9-3.6) | 3.2 (2.9-3.6) | 3.2 (3.0-3.5) | 1.5 (1.2-1.7) | 1.0 (0.9-1.2) |
| Other gliomas | 1.0 | 0.8 | 0.7 | 0.9 | 0.7 (0.5-0.9) | 1.0 (0.8-1.2) | 0.9 (0.7-1.0) | 1.3 (0.9-1.8) | 1.4 (1.0-2.0) |
| Other specified | 0.2 | 0.2 | 0.1 | 0.2 | 0.2 (0.1-0.3) | 0.2 (0.1-0.3) | 0.2 (0.1-0.3) | 1.4 (0.7-2.7) | 1.2 (0.6-2.4) |
| Unspecified CNS | 3.5 | 2.8 | 1.6 | 2.6 | 0.9 (0.7-1.1) | 4.3 (3.9-4.8) | 2.7 (2.4-2.9) | 1.1 (0.9-1.4) | 4.8 (3.9-6.0) |
| **NEUROBLASTOMA** | **8.5** | **2.8** | **0.9** | **4.0** | **3.8 (3.4-4.2)** | **4.3 (3.9-4.7)** | **4.1 (3.8-4.4)** | **0.9 (0.8-1.1)** | 1.1 (1.0-1.3) |
| Neuroblastoma and ganglioneuroblastoma | 8.4 | 2.7 | 0.8 | 4.0 | 3.8 (3.3-4.2) | 4.2 (3.8-4.7) | 4.0 (3.7-4.3) | 0.9 (0.8-1.1) | 1.1 (1.0-1.3) |
| peripheral nervous | 0.1 | 0.0 | 0.1 | 0.1 | 0.1 (0.0-0.1) | 0.1 (0.0-0.1) | 0.1 (0.0-0.1) | 0.6 (0.2-1.8) | 1.1 (0.4-3.3) |
| **RETINOBLASTOMA** | **6.0** | **0.9** | **0.1** | **2.3** | **2.8 (2.4-3.1)** | **2.0 (1.7-2.3)** | **2.4 (2.1-2.6)** | **1.1 (0.9-1.3)** | 0.7 (0.6-0.9) |
| **RENAL TUMOURS** | **9.9** | **3.5** | **0.9** | **4.8** | **4.8 (4.3-5.2)** | **4.9 (4.4-5.3)** | **4.8 (4.5-5.1)** | **1.0 (0.9-1.2)** | 1.0 (0.9-1.2) |
| Nephroblastoma | 7.8 | 3.0 | 0.6 | 3.8 | 4.1 (3.7-4.5) | 3.7 (3.3-4.1) | 3.9 (3.6-4.2) | 0.9 (0.8-1.1) | 0.9 (0.8-1.0) |
| Renal carcinomas | 1.4 | 0.2 | 0.2 | 0.6 | 0.6 (0.4-0.8) | 0.6 (0.5-0.8) | 0.6 (0.5-0.7) | 1.1 (0.7-1.5) | 1.1 (0.7-1.6) |
| Unspecified | 0.6 | 0.2 | 0.1 | 0.3 | 0.1 (0.0-0.1) | 0.6 (0.4-0.7) | 0.3 (0.2-0.4) | 1.9 (1.1-3.2) | 8.2 (4.1-16.5) |
| **HEPATIC TUMOURS** | **4.7** | **1.0** | **0.8** | **2.2** | **1.5 (1.2-1.7)** | **2.8 (2.5-3.2)** | **2.2 (1.9-2.4)** | **1.4 (1.1-1.7)** | 1.9 (1.5-2.3) |
| Hepatoblastoma | 2.4 | 0.3 | 0.2 | 1.0 | 0.7 (0.6-0.9) | 1.2 (1.0-1.4) | 1.0 (0.9-1.1) | 1.4 (1.0-1.9) | 1.6 (1.2-2.2) |
| Hepatic carcinomas | 1.0 | 0.3 | 0.4 | 0.6 | 0.5 (0.4-0.7) | 0.6 (0.5-0.8) | 0.6 (0.5-0.7) | 1.4 (0.9-2.1) | 1.3 (0.9-1.8) |
| Unspecified | 1.2 | 0.3 | 0.2 | 0.6 | 0.2 (0.1-0.3) | 0.9 (0.7-1.1) | 0.6 (0.5-0.7) | 1.6 (1.1-2.3) | 4.7 (3.0-7.4) |

Table S4 (Continue). Age-specific incidence rate, ASIRs (per million person-years) for ICCC groups and subgroups

| **ICCC group** | **Age at Diagnosis (Age-Specific Rate)** | | | | **ASIR for Five-year period of diagnosis (95% CI)** | | **Total ASIR** | **SRR (95% CI)** | |
| --- | --- | --- | --- | --- | --- | --- | --- | --- | --- |
|  | **0-4** | **5-9** | **10-14** | **0-14** | **2005-2009** | **2010-2014** | **2005-2014** | **Male to Female** | **2010-2014 to 2005-2009** |
| **BONE TUMOURS** | **2.0** | **4.1** | **9.6** | **5.3** | **5.2 (4.7-5.7)** | **5.2 (4.7-5.7)** | **5.2 (4.9-5.5)** | **1.0 (0.9-1.1)** | 1.0 (0.9-1.1) |
| Osteosarcomas | 0.3 | 1.8 | 5.8 | 2.7 | 2.8 (2.4-3.1) | 2.4 (2.1-2.8) | 2.6 (2.4-2.9) | 0.9 (0.7-1.0) | 0.9 (0.7-1.1) |
| Chondrosarcomas | 0.2 | 0.1 | 0.2 | 0.2 | 0.2 (0.1-0.3) | 0.1 (0.1-0.2) | 0.2 (0.1-0.2) | 1.1 (0.5-2.3) | 0.8 (0.4-1.7) |
| Ewing and related | 0.7 | 1.6 | 2.3 | 1.5 | 1.7 (1.4-2.0) | 1.3 (1.1-1.6) | 1.5 (1.3-1.7) | 0.9 (0.7-1.2) | 0.8 (0.6-1.0) |
| Other specified | 0.1 | 0.2 | 0.2 | 0.2 | 0.2 (0.1-0.3) | 0.2 (0.1-0.3) | 0.2 (0.1-0.2) | 1.5 (0.7-3.0) | 0.9 (0.5-1.9) |
| Unspecified | 0.8 | 0.4 | 1.0 | 0.7 | 0.4 (0.3-0.5) | 1.1 (0.9-1.3) | 0.7 (0.6-0.9) | 1.4 (1.0-2.0) | 2.8 (2.0-4.1) |
| **SOFT TISSUE SARCOMA** | **7.3** | **4.2** | **5.6** | **5.7** | **5.7 (5.2-6.2)** | **5.7 (5.2-6.2)** | **5.7 (5.4-6.1)** | **1.0 (0.9-1.2)** | 1.0 (0.9-1.1) |
| Rhabdomyosarcomas | 3.2 | 2.0 | 1.4 | 2.2 | 2.3 (2.0-2.7) | 2.1 (1.8-2.3) | 2.2 (2.0-2.5) | 1.0 (0.8-1.3) | 0.9 (0.7-1.1) |
| Fibrosarcomas and other | 0.7 | 0.2 | 0.5 | 0.5 | 0.4 (0.3-0.6) | 0.5 (0.4-0.6) | 0.5 (0.4-0.6) | 1.1 (0.7-1.7) | 1.0 (0.7-1.6) |
| Kaposi sarcoma | 0.2 | 0.0 | 0.0 | 0.1 | 0.1 (0.0-0.1) | 0.1 (0.0-0.2) | 0.1 (0.0-0.1) | 1.1 (0.4-3.0) | 1.4 (0.5-3.9) |
| Other specified | 2.3 | 1.2 | 2.4 | 2.0 | 1.7 (1.5-2.0) | 2.2 (1.9-2.5) | 2.0 (1.8-2.2) | 1.0 (0.8-1.3) | 1.3 (1.0-1.6) |
| Unspecified | 0.9 | 0.7 | 1.3 | 1.0 | 1.1 (0.9-1.3) | 0.9 (0.7-1.1) | 1.0 (0.9-1.2) | 1.0 (0.8-1.4) | 0.8 (0.6-1.1) |
| **GERM CELL TUMOURS** | **6.1** | **1.0** | **2.3** | **3.1** | **3.2 (2.8-3.6)** | **3.0 (2.7-3.4)** | **3.1 (2.9-3.4)** | **0.7 (0.6-0.8)** | 0.9 (0.8-1.1) |
| Intracranial and intraspinal | 0.2 | 0.1 | 0.2 | 0.2 | 0.2 (0.1-0.2) | 0.2 (0.1-0.3) | 0.2 (0.1-0.2) | 0.6 (0.3-1.2) | 1.1 (0.6-2.3) |
| Malignant extracranial and extragonadal | 2.0 | 0.2 | 0.3 | 0.9 | 0.8 (0.6-1.0) | 0.9 (0.7-1.1) | 0.9 (0.8-1.0) | 0.4 (0.3-0.6) | 1.1 (0.8-1.5) |
| Malignant gonadal germ cell | 2.7 | 0.5 | 1.5 | 1.6 | 1.9 (1.6-2.2) | 1.2 (1.0-1.4) | 1.6 (1.4-1.8) | 1.2 (0.9-1.5) | 0.6 (0.5-0.8) |
| Gonadal carcinomas | 0.6 | 0.1 | 0.1 | 0.3 | 0.2 (0.1-0.3) | 0.3 (0.2-0.4) | 0.3 (0.2-0.3) | 0.0 (0.0-0.1) | 1.8 (1.0-3.2) |
| Other and unspecified gonadal | 0.6 | 0.2 | 0.1 | 0.3 | 0.2 (0.1-0.2) | 0.4 (0.3-0.6) | 0.3 (0.2-0.4) | 0.6 (0.3-1.0) | 2.8 (1.6-5.1) |
| **OTHER MALIGNANT & CARCINOMA & MELANOMA** | **49.7** | **5.2** | **6.9** | **20.8** | **18.2 (17.2-19.1)** | **23.2 (22.3-24.2)** | **20.8 (20.2-21.5)** | **1.3 (1.3-1.4)** | 1.6 (1.6-1.7) |
| Adrenocortical | 0.1 | 0.0 | 0.0 | 0.1 | 0.1 (0.0-0.1) | 0.1 (0.0-0.1) | 0.1 (0.0-0.1) | 0.2 (0.1-1.0) | 0.9 (0.3-3.2) |
| Thyroid | 1.9 | 0.3 | 2.4 | 1.6 | 1.4 (1.1-1.6) | 1.7 (1.4-1.9) | 1.5 (1.4-1.7) | 0.3 (0.3-0.4) | 1.2 (1.0-1.5) |
| Nasopharyngeal | 0.1 | 0.1 | 0.3 | 0.2 | 0.2 (0.1-0.3) | 0.1 (0.1-0.2) | 0.2 (0.1-0.3) | 1.7 (0.9-3.4) | 0.6 (0.3-1.1) |
| Malignant melanomas | 1.0 | 0.1 | 0.2 | 0.5 | 0.4 (0.3-0.6) | 0.5 (0.4-0.6) | 0.5 (0.4-0.6) | 1.1 (0.7-1.6) | 1.2 (0.8-1.9) |
| Skin carcinomas | 0.6 | 1.3 | 1.1 | 1.0 | 0.9 (0.7-1.1) | 1.2 (1.0-1.4) | 1.0 (0.9-1.2) | 1.4 (1.0-1.9) | 2.5 (2.3-2.7) |
| Other & unspecified | 45.9 | 3.3 | 2.8 | 17.5 | 15.2 (14.4-16.0) | 19.7 (18.8-20.6) | 17.6 (17.0-18.2) | 1.2 (1.1-1.3) | 1.3 (1.2-1.4) |
| **OTHER & UNSPECIFIED** | **19.4** | **6.3** | **5.0** | **10.3** | **6.1 (5.6-6.6)** | **14.1 (13.4-14.9)** | **10.3 (9.8-10.8)** | **1.4 (1.2-1.5)** | 2.3 (2.1-2.5) |
| Other specified | 0.3 | 0.1 | 0.0 | 0.1 | 0.1 (0.1-0.2) | 0.1 (0.1-0.2) | 0.1 (0.1-0.2) | 1.9 (0.8-4.3) | 1.0 (0.5-2.3) |
| Other unspecified | 19.1 | 6.3 | 5.0 | 10.1 | 6.0 (5.5-6.5) | 14.0 (13.2-14.8) | 10.2 (9.7-10.7) | 1.3 (1.2-1.5) | 2.3 (2.1-2.5) |
| **Total** | **179.4** | **88.8** | **78.7** | **115.9** | **103.0 (100.9-105.2)** | **127.8 (125.5-130.1)** | **116.2 (114.6-117.7)** | **1.2 (1.2-1.3)** | **1.3 (1.3-1.4)** |

Table S5. Joinpoint regression analysis of time trends in ASIRs (per million person-years) of childhood cancers in Iran, 2005–2014.

| **Subgroup** | **Sex** | **No. of Joinpoints** | **AAPC (95% CI)** | **P value** |
| --- | --- | --- | --- | --- |
| **LEUKAEMIA** |  |  |  |  |
|  | Female | 0 | 2.35 (-0.30, 5.10) | 0.089 |
|  | Male | 0 | 1.77 (-1.02, 4.60) | 0.237 |
|  | Total | 0 | 2.00 (-0.33, 4.35) | 0.102 |
| Lymphoid | Female | 0 | 4.76 (-1.29, 10.98) | 0.117 |
| Lymphoid | Male | 0 | 2.88 (-3.00, 9.05) | 0.368 |
| Lymphoid | Total | 0 | 3.64 (-1.62, 9.15) | 0.190 |
| Acute myeloid | Female | 0 | 1.56 (-8.04, 11.98) | 0.768 |
| Acute myeloid | Male | 0 | 0.15 (-6.56, 7.43) | 0.977 |
| Acute myeloid | Total | 0 | 0.69 (-6.51, 8.35) | 0.850 |
| CMD | Female | 0 | -6.01 (-16.25, 5.15) | 0.246 |
| CMD | Male | 0 | 1.46 (-19.85, 28.24) | 0.911 |
| CMD | Total | 0 | -3.39 (-12.17, 5.86) | 0.405 |
| Unspecified and other specified | Female | 1 | 28.64* (7.96, 46.59) | 0.001 |
| Unspecified and other specified | Male | 1 | 29.38* (3.65, 53.77) | 0.014 |
| Unspecified and other specified | Total | 1 | 29.14* (3.34, 53.60) | 0.016 |
| **LYMPHOMA & RELATED** |  |  |  |  |
|  | Female | 0 | 0.49 (-1.28, 2.28) | 0.541 |
|  | Male | 0 | -0.01 (-2.42, 2.40) | 0.974 |
|  | Total | 0 | 0.14 (-2.56, 2.85) | 0.918 |
| Hodgkin | Female | 1 | -5.42* (-9.41, -0.89) | 0.020 |
| Hodgkin | Male | 0 | -1.96 (-6.95, 3.17) | 0.359 |
| Hodgkin | Total | 1 | -2.21* (-4.99, -0.37) | 0.022 |
| Non-Hodgkin except BL | Female | 1 | 9.35 (-4.89, 23.76) | 0.152 |
| Non-Hodgkin except BL | Male | 0 | 3.37 (-1.56, 8.40) | 0.196 |
| Non-Hodgkin except BL | Total | 0 | 4.75* (0.36, 9.26) | 0.034 |
| Burkitt (BL) | Female | 1 | -0.98 (-8.81, 9.13) | 0.898 |
| Burkitt (BL) | Male | 0 | -4.68 (-11.34, 2.46) | 0.182 |
| Burkitt (BL) | Total | 0 | -3.32 (-9.29, 3.05) | 0.284 |
| Unspecified | Female | 0 | 5.57 (-8.45, 21.46) | 0.439 |
| Unspecified | Male | 0 | 1.00 (-9.13, 11.76) | 0.844 |
| Unspecified | Total | 0 | 2.03 (-7.72, 12.61) | 0.681 |
| **CNS NEOPLASMS** |  |  |  |  |
|  | Female | 1 | 5.78* (1.59, 9.64) | 0.004 |
|  | Male | 1 | 4.24* (2.36, 6.02) | < 0.001 |
|  | Total | 1 | 4.91* (1.27, 8.13) | 0.004 |
| Ependymoma and choroid plexus tumor | Female | 0 | 3.30 (-9.43, 17.40) | 0.573 |
| Ependymoma and choroid plexus tumor | Male | 0 | 3.59 (-7.02, 15.39) | 0.493 |
| Ependymoma and choroid plexus tumor | Total | 0 | 3.59 (-2.35, 9.77) | 0.241 |
| Astrocytoma | Female | 1 | 9.95* (3.30, 16.82) | 0.002 |
| Astrocytoma | Male | 1 | 6.10* (3.14, 8.83) | < 0.001 |
| Astrocytoma | Total | 1 | 7.89* (4.61, 11.44) | < 0.001 |
| Intracranial and intraspinal embryonal tumors | Female | 0 | 3.20 (-2.46, 9.08) | 0.263 |
| Intracranial and intraspinal embryonal tumors | Male | 0 | -0.05 (-4.06, 4.06) | 0.967 |
| Intracranial and intraspinal embryonal tumors | Total | 0 | 1.23 (-3.95, 6.56) | 0.557 |
| Other gliomas | Female | 0 | 8.75 (-8.47, 28.60) | 0.322 |
| Other gliomas | Male | 0 | 6.67 (-4.09, 18.53) | 0.236 |
| Other gliomas | Total | 0 | 7.05 (-0.72, 14.87) | 0.078 |
| Other specified | Total | 1 | 0.29 (-15.20, 17.92) | 0.949 |
| **NEUROBLASTOMA** |  |  |  |  |
|  | Female | 0 | -0.93 (-5.04, 3.40) | 0.665 |
|  | Male | 1 | -1.26 (-4.37, 2.64) | 0.483 |
|  | Total | 0 | -0.67 (-4.19, 2.96) | 0.707 |
| Neuroblastoma and ganglioneuroblastoma | Female | 0 | 0.14 (-8.23, 9.30) | 0.989 |
| Neuroblastoma and ganglioneuroblastoma | Male | 1 | -0.66 (-6.51, 7.01) | 0.935 |
| Neuroblastoma and ganglioneuroblastoma | Total | 0 | 0.59 (-6.14, 7.74) | 0.899 |
| **V. Retinoblastoma** |  |  |  |  |
|  | Female | 0 | -4.26* (-7.44, -1.09) | 0.012 |
|  | Male | 0 | -4.40* (-7.87, -0.87) | 0.014 |
|  | Total | 0 | -4.27* (-6.31, -2.19) | < 0.001 |
| **RENAL TUMOURS** |  |  |  |  |
|  | Female | 0 | -0.49 (-2.65, 1.70) | 0.588 |
|  | Male | 0 | 0.47 (-2.64, 3.67) | 0.789 |
|  | Total | 0 | -0.02 (-1.58, 1.58) | 0.966 |
| Nephroblastoma | Female | 1 | 0.30 (-4.45, 3.34) | 0.965 |
| Nephroblastoma | Male | 0 | -1.13 (-9.30, 7.66) | 0.754 |
| Nephroblastoma | Total | 0 | -1.31 (-7.58, 5.17) | 0.631 |
| **HEPATIC TUMOURS** |  |  |  |  |
|  | Female | 1 | 10.25* (8.40, 12.14) | < 0.001 |
|  | Male | 1 | 9.58* (5.12, 13.63) | < 0.001 |
|  | Total | 1 | 9.41* (6.09, 12.63) | < 0.001 |
| Hepatoblastoma | Female | 0 | 8.55 (-0.91, 18.78) | 0.074 |
| Hepatoblastoma | Male | 0 | 11.82* (0.87, 24.15) | 0.036 |
| Hepatoblastoma | Total | 0 | 10.55* (4.09, 17.24) | < 0.001 |
| Hepatic carcinomas | Male | 1 | 25.79* (13.77, 39.45) | < 0.001 |
| Hepatic carcinomas | Total | 1 | 31.81* (20.94, 45.04) | < 0.001 |
| **BONE TUMOURS** |  |  |  |  |
|  | Female | 1 | 1.54 (-0.58, 3.16) | 0.146 |
|  | Male | 0 | 0.62 (-2.57, 3.85) | 0.698 |
|  | Total | 1 | 1.28 (-0.77, 3.13) | 0.203 |
| Osteosarcomas | Female | 1 | -0.41 (-8.28, 6.05) | 0.809 |
| Osteosarcomas | Male | 0 | -2.63 (-11.40, 6.75) | 0.543 |
| Osteosarcomas | Total | 1 | -0.83 (-9.56, 6.60) | 0.695 |
| Ewing and related | Female | 1 | -2.09 (-5.70, 1.24) | 0.238 |
| Ewing and related | Male | 0 | -6.76 (-14.74, 1.80) | 0.116 |
| Ewing and related | Total | 1 | -2.18 (-8.08, 1.67) | 0.200 |
| Other specified | Total | 1 | 7.89 (-8.03, 23.44) | 0.378 |
| Unspecified | Male | 1 | 31.63* (4.51, 61.31) | 0.012 |
| Unspecified | Total | 0 | 28.00* (11.47, 47.07) | 0.001 |
| **SOFT TISSUE SARCOMA** |  |  |  |  |
|  | Female | 0 | -0.58 (-3.88, 2.77) | 0.655 |
|  | Male | 0 | 0.90 (-0.75, 2.56) | 0.282 |
|  | Total | 0 | 0.17 (-1.50, 1.87) | 0.871 |
| Rhabdomyosarcomas | Female | 0 | -6.18 (-12.95, 1.05) | 0.091 |
| Rhabdomyosarcomas | Male | 0 | 2.75 (-1.54, 7.16) | 0.214 |
| Rhabdomyosarcomas | Total | 0 | -1.60 (-6.14, 3.19) | 0.510 |
| Fibrosarcomas and other | Female | 0 | 8.77 (-5.42, 25.36) | 0.242 |
| Fibrosarcomas and other | Male | 0 | -2.98 (-20.12, 17.52) | 0.699 |
| Fibrosarcomas and other | Total | 0 | 1.93 (-12.10, 17.54) | 0.777 |
| Other specified | Female | 0 | 3.65 (-7.00, 15.34) | 0.512 |
| Other specified | Male | 0 | 6.67 (-3.30, 17.90) | 0.200 |
| Other specified | Total | 0 | 5.20 (-3.21, 14.30) | 0.238 |
| Unspecified | Female | 0 | -2.12 (-11.48, 8.13) | 0.641 |
| Unspecified | Male | 0 | -3.59 (-14.48, 8.49) | 0.495 |
| Unspecified | Total | 0 | -2.83 (-11.09, 5.89) | 0.430 |
| **GERM CELL TUMOURS** |  |  |  |  |
|  | Female | 1 | 1.36 (-1.63, 4.04) | 0.294 |
|  | Male | 0 | -1.96 (-5.29, 1.40) | 0.237 |
|  | Total | 1 | -1.59 (-4.94, 2.69) | 0.310 |
| Malignant extracranial and extragonadal | Female | 0 | 0.96 (-13.18, 16.87) | 0.905 |
| Malignant extracranial and extragonadal | Male | 0 | 1.45 (-14.77, 20.52) | 0.861 |
| Malignant extracranial and extragonadal | Total | 0 | -0.15 (-8.65, 9.11) | 0.967 |
| Malignant gonadal germ cell | Female | 0 | -8.26 (-16.47, 0.68) | 0.067 |
| Malignant gonadal germ cell | Male | 1 | -12.60* (-20.11, -3.37) | 0.014 |
| Malignant gonadal germ cell | Total | 1 | -8.25* (-13.96, -2.47) | 0.004 |
| **OTHER MALIGNANT & CARCINOMA & MELANOMA** |  |  |  |  |
|  | Female | 1 | 10.72 (-0.91, 23.75) | 0.066 |
|  | Male | 1 | 16.54* (6.42, 28.03) | 0.002 |
|  | Total | 1 | 11.85 (-0.83, 25.99) | 0.060 |
| Thyroid | Female | 0 | 11.10 (-0.10, 22.87) | 0.054 |
| Thyroid | Male | 0 | 1.99 (-10.99, 16.58) | 0.762 |
| Thyroid | Total | 1 | 10.84* (2.53, 19.37) | 0.012 |
| Nasopharyngeal | Total | 0 | -8.04 (-19.02, 4.22) | 0.208 |
| Skin carcinomas | Male | 1 | 19.48* (4.72, 36.18) | 0.011 |
| Skin carcinomas | Total | 1 | 23.77* (17.79, 30.34) | < 0.001 |
| Other & unspecified | Female | 1 | 31.53 (-2.91, 76.92) | 0.068 |
| Other & unspecified | Male | 1 | 41.78* (0.06, 98.64) | 0.050 |
| Other & unspecified | Total | 1 | 36.11* (0.84, 83.34) | 0.047 |
| **OTHER & UNSPECIFIED** |  |  |  |  |
|  | Female | 0 | 10.19* (1.47, 19.60) | 0.025 |
|  | Male | 0 | 9.36* (0.66, 18.58) | 0.036 |
|  | Total | 0 | 9.70* (1.06, 18.96) | 0.031 |
| Other unspecified | Female | 0 | 22.83* (3.80, 45.06) | 0.020 |
| Other unspecified | Male | 0 | 21.05* (2.11, 43.20) | 0.034 |
| Other unspecified | Total | 0 | 21.80* (3.23, 43.10) | 0.022 |
| **Total** |  |  |  |  |
|  | Female | 1 | 3.63* (0.44, 6.65) | 0.033 |
|  | Male | 1 | 3.31* (0.20, 6.26) | 0.038 |
|  | Total | 1 | 3.47 (0.00, 6.78) | 0.050 |

Note: Joinpoint regression analysis was applied to evaluate temporal trends in age-standardized incidence rates (ASIRs, per million person-years) of childhood cancers in Iran during 2005–2014, using the Joinpoint Regression Program (Version 5.1.1, National Cancer Institute, USA). The model identifies significant changes (“joinpoints”) in log-linear trends over time and estimates the annual percentage change (APC) for each segment and the average annual percentage change (AAPC) across the entire period. AAPCs with 95% confidence intervals (CIs) and p-values are presented separately for each ICCC subgroup and by sex (female, male, total). Positive AAPC values indicate increasing trends, while negative values indicate decreasing trends. Asterisks (*) denote statistically significant changes (p < 0.05). Abbreviations: AAPC, average annual percentage change; ASIR, age-standardized incidence rate; CI, confidence interval; ICCC, International Classification of Childhood Cancer.

Table S6. Projected ASIR (per million person-years) of Childhood Cancer by Sex, 2015–2020

| **Year** | **Total** | **Male** | **Female** |
| --- | --- | --- | --- |
| 2015 | 139.92 | 153.40 | 125.88 |
| 2016 | 145.89 | 160.04 | 131.15 |
| 2017 | 151.85 | 166.67 | 136.42 |
| 2018 | 157.82 | 173.31 | 141.69 |
| 2019 | 163.78 | 179.95 | 146.96 |
| 2020 | 169.75 | 186.59 | 152.23 |

Note: Projections were generated using structural time series (STS) models with a stochastic trend component, fitted separately to the World Health Organization (WHO) standardized annual age-standardized incidence rates (ASIRs) for males, females, and the total population (2005–2014). The STS model decomposes each series into a deterministic trend ($m_{t}$) and a random irregular component ($Y_{t}$), and forecasts were produced through 2020 with 95% confidence intervals. The male-to-female ratio was calculated annually from the projected values. *(Brockwell PJ, Davis RA. Introduction to Time Series and Forecasting. Springer New York; 2013.)*

Table S7. ASIR (per million person-years) of childhood cancer in Iran by province, sex, and period (2005–2009, 2010–2014, and 2005–2014).

| **Province** | **Overall** | | | **Male** | | | **Female** | | |
| --- | --- | --- | --- | --- | --- | --- | --- | --- | --- |
|  | **2005-2009** | **2010-2014** | **2005-2014** | **2005-2009** | **2010-2014** | **2005-2014** | **2005-2009** | **2010-2014** | **2005-2014** |
| Alborz | 118.62 | 117.47 | 117.96 | 130.54 | 130.34 | 130.38 | 106.08 | 103.96 | 104.93 |
| Ardabil | 83.94 | 259.59 | 174.50 | 83.66 | 324.35 | 207.36 | 84.23 | 190.34 | 139.51 |
| Bushehr | 127.96 | 111.99 | 118.90 | 142.10 | 132.63 | 135.91 | 113.12 | 90.49 | 101.06 |
| Chahar Mahaal and Bakhtiari | 69.48 | 83.80 | 77.02 | 66.69 | 84.26 | 76.66 | 72.38 | 83.09 | 77.32 |
| East Azarbaijan | 122.80 | 102.45 | 112.12 | 137.16 | 106.91 | 121.12 | 107.64 | 97.75 | 102.59 |
| Fars | 150.90 | 181.82 | 167.84 | 169.11 | 203.35 | 188.02 | 131.78 | 159.20 | 146.62 |
| Ghazvin | 61.94 | 86.30 | 74.60 | 64.67 | 96.29 | 80.88 | 59.08 | 75.72 | 67.93 |
| Gilan | 62.15 | 131.75 | 96.82 | 74.15 | 134.44 | 104.33 | 49.61 | 128.94 | 88.98 |
| Golestan | 52.96 | 54.42 | 54.19 | 65.00 | 57.18 | 61.51 | 40.44 | 51.55 | 46.59 |
| Hamedan | 73.32 | 109.71 | 91.23 | 81.10 | 112.39 | 96.54 | 65.07 | 106.84 | 85.59 |
| Hormozgan | 66.16 | 74.35 | 70.31 | 77.40 | 85.89 | 81.69 | 54.42 | 62.30 | 58.42 |
| Ilam | 47.08 | 47.01 | 46.16 | 41.40 | 49.22 | 43.53 | 53.08 | 44.66 | 48.96 |
| Isfahan | 72.59 | 129.36 | 102.01 | 76.93 | 141.03 | 110.02 | 68.05 | 117.12 | 93.61 |
| Kerman | 89.58 | 112.73 | 101.90 | 113.45 | 124.47 | 119.91 | 64.63 | 100.45 | 83.11 |
| Kermanshah | 51.33 | 49.11 | 50.09 | 56.51 | 44.46 | 50.32 | 45.83 | 54.10 | 49.86 |
| Khuzestan | 138.64 | 149.87 | 144.63 | 161.45 | 169.72 | 166.03 | 114.60 | 129.00 | 122.10 |
| Kohgiluyeh and Boyer-Ahmad | 94.54 | 165.81 | 130.94 | 119.44 | 152.96 | 137.52 | 68.44 | 179.38 | 124.05 |
| Kurdistan | 87.79 | 81.68 | 84.95 | 98.93 | 95.12 | 96.66 | 76.14 | 67.63 | 72.66 |
| Lorestan | 51.96 | 128.47 | 91.70 | 53.07 | 144.71 | 100.58 | 50.77 | 111.09 | 82.23 |
| Markazi | 81.50 | 182.88 | 133.28 | 76.13 | 193.84 | 135.94 | 87.12 | 171.45 | 130.51 |
| Mazandaran | 134.31 | 147.00 | 140.86 | 142.69 | 165.52 | 154.07 | 125.57 | 127.80 | 127.12 |
| North Khorasan | 58.32 | 82.41 | 70.34 | 64.44 | 91.63 | 78.37 | 51.92 | 72.64 | 61.86 |
| Qom | 51.98 | 57.03 | 54.48 | 46.67 | 61.15 | 53.89 | 57.59 | 52.75 | 55.11 |
| Razavi Khorasan | 96.44 | 134.37 | 116.92 | 107.21 | 142.00 | 126.04 | 85.19 | 126.41 | 107.38 |
| Semnan | 84.73 | 101.29 | 93.48 | 110.44 | 115.06 | 112.81 | 57.92 | 87.11 | 73.45 |
| Sistan and Baluchestan | 53.95 | 80.79 | 67.97 | 54.49 | 89.10 | 72.50 | 53.36 | 72.10 | 63.23 |
| Southern Khorasan | 83.33 | 78.01 | 81.11 | 76.28 | 106.07 | 92.54 | 90.78 | 48.58 | 69.12 |
| Tehran | 118.62 | 117.47 | 117.96 | 130.54 | 130.34 | 130.38 | 106.08 | 103.96 | 104.93 |
| West Azarbaijan | 88.61 | 66.20 | 77.58 | 95.05 | 69.97 | 82.96 | 81.82 | 62.18 | 71.90 |
| Yazd | 126.18 | 180.39 | 155.77 | 178.30 | 174.51 | 177.31 | 71.62 | 186.59 | 133.38 |
| Zanjan | 61.36 | 109.10 | 85.19 | 64.33 | 138.50 | 101.98 | 58.25 | 78.25 | 67.56 |

Note: ASIR values represent age-standardized incidence rates of childhood cancers (ages 0–14 years) per million person-years, calculated for each Iranian province across two five-year intervals (2005–2009 and 2010–2014) and for the combined decade (2005–2014). Rates are stratified by sex (total, male, and female).


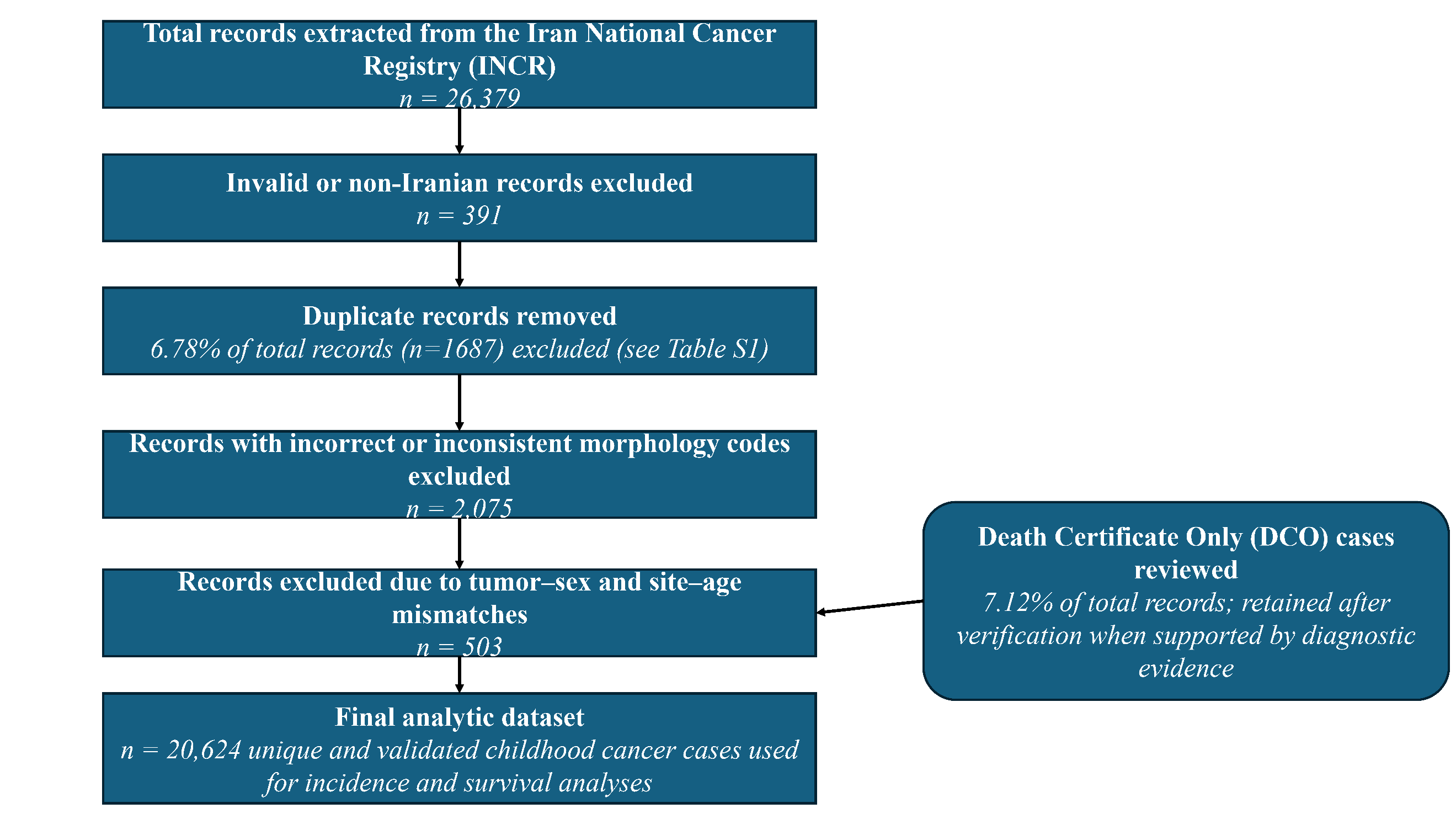


Figure S1. Flowchart illustrating the data extraction, cleaning, and validation process of childhood cancer cases recorded in the Iran National Cancer Registry (INCR), 2005–2014.


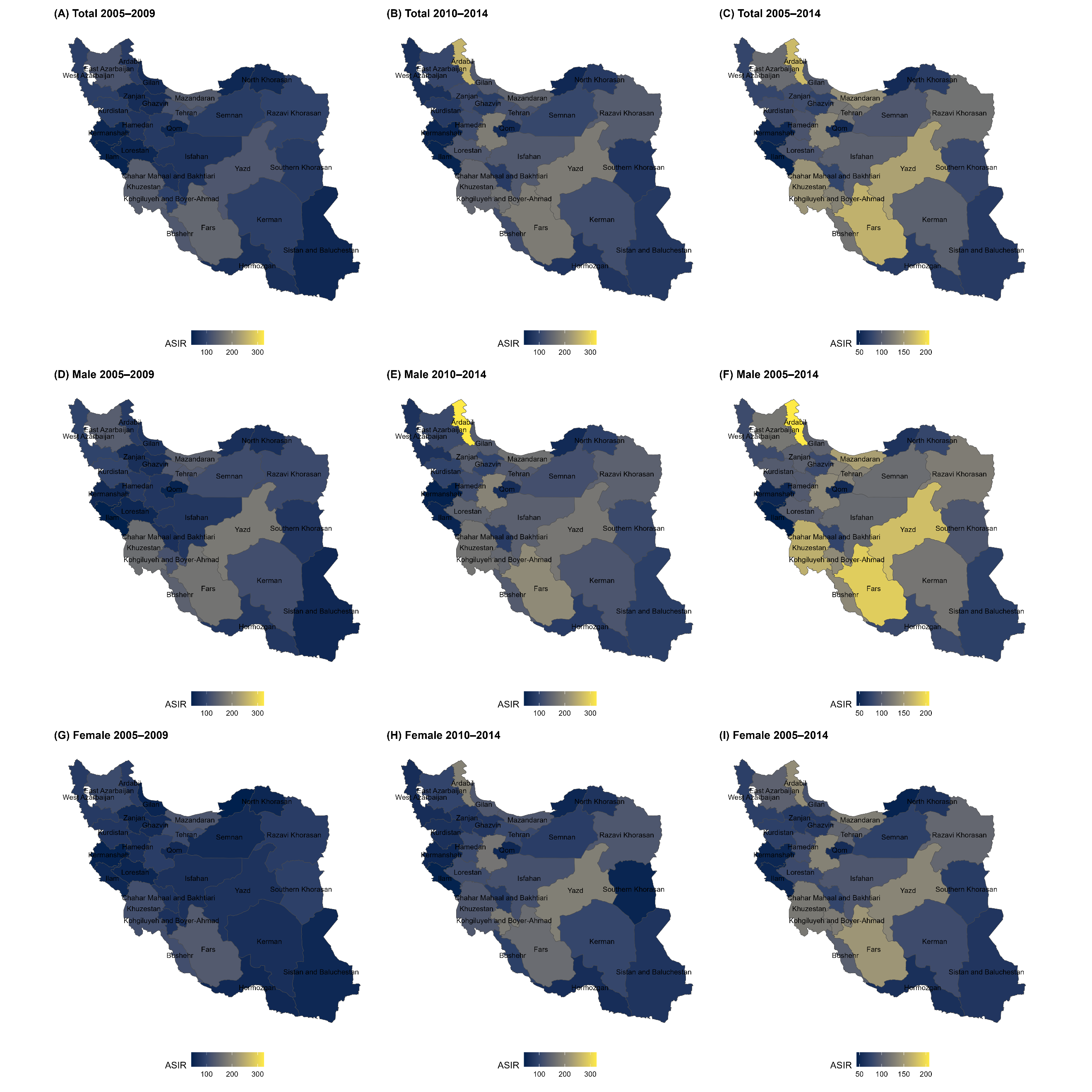


Figure S2. Spatial Distribution of Childhood Cancer ASIR in Iran by Sex and Period (2005–2009, 2010–2014, and 2005–2014) [*Panels (A–C) show total cases, (D–F) male cases, and (G–I) female cases for the periods 2005–2009, 2010–2014, and 2005–2014, respectively. To ensure valid spatial comparisons, ASIR maps for 2005–2009 and 2010–2014 share a common color scale range, while the overall 2005–2014 maps are displayed using an independent range due to their broader value distribution. Yellow shades indicate provinces with higher ASIR values, and darker tones represent lower rates.]*
